# Agent-Based Models Predict Emergent Behavior of Heterogeneous Cell Populations in Dynamic Microenvironments

**DOI:** 10.3389/fbioe.2020.00249

**Published:** 2020-06-11

**Authors:** Jessica S. Yu, Neda Bagheri

**Affiliations:** ^1^Chemical and Biological Engineering, Northwestern University, Evanston, IL, United States; ^2^Northwestern Institute on Complex Systems, Northwestern University, Evanston, IL, United States; ^3^Center for Synthetic Biology, Northwestern University, Evanston, IL, United States; ^4^Biology, University of Washington, Seattle, WA, United States; ^5^Chemical Engineering, University of Washington, Seattle, WA, United States

**Keywords:** agent-based model, cell population dynamics, computational modeling, emergent behavior, microenvironment

## Abstract

Computational models are most impactful when they explain and characterize biological phenomena that are non-intuitive, unexpected, or difficult to study experimentally. Countless equation-based models have been built for these purposes, but we have yet to realize the extent to which rules-based models offer an intuitive framework that encourages computational and experimental collaboration. We develop ARCADE, a multi-scale agent-based model to interrogate emergent behavior of heterogeneous cell agents within dynamic microenvironments and demonstrate how complexity of intracellular metabolism and signaling modules impacts emergent dynamics. We perform *in silico* case studies on context, competition, and heterogeneity to demonstrate the utility of our model for gaining computational and experimental insight. Notably, there exist (i) differences in emergent behavior between colony and tissue contexts, (ii) linear, non-linear, and multimodal consequences of parameter variation on competition in simulated co-cultures, and (iii) variable impact of cell and population heterogeneity on emergent outcomes. Our extensible framework is easily modified to explore numerous biological systems, from tumor microenvironments to microbiomes.

## 1. Introduction

Computational models are *in silico* tools used to represent a system or phenomenon of interest, with wide ranging applications in both experimental and clinical settings (Winslow et al., 2012; Brodland, 2015). With increasingly high resolution and high throughput experimental techniques, computational models become essential for summarizing, integrating, and exploring high dimensional data sets. While reactive data-driven computational models are ubiquitous—from simple, single equations fitting population level aggregate metrics to more complex differential equation systems—we have yet to realize the full impact of proactive models to provide *de novo* insights in cases where experimental techniques are inadequate or insufficient. Computational modeling has the potential to overcome experimental limitations in three major areas: spatial and temporal resolution, intra- and intercellular heterogeneity, and environmental context.

First, biological systems exhibit spatial and temporal variation as observed in cell fate commitment during development, cell state commitment in pattern formation, and circadian-regulated gene expression (Zernicka-Goetz, 2004; Zhang et al., 2014; Manukyan et al., 2017). Models that are able to capture such behavior with high temporal and spatial resolution allow rigorous systems analysis and hypothesis testing that is often not possible experimentally.

Second, biological systems are highly heterogeneous, both between and within cell types. The immune system, for example, is composed of a number of different cell types, each with its own unique role. Studies have demonstrated remarkable phenotypic variation within tumor cell populations (Dagogo-Jack and Shaw, 2017) and highly diverse species within microbial communities (Eckburg, 2005). Homogeneous experimental systems fail to account for this diversity and its role in shaping behavior. In addition, heterogeneity within a computational model can be measured and tuned precisely whereas the same quantification and control in an experimental setting is much more difficult.

Finally, biological systems exist within diverse environmental contexts. The tumor microenvironment, for instance, has received significant attention as a major contributor to disease prognosis (Balkwill et al., 2012; Quail and Joyce, 2013). Cells cultured in 2D vs. 3D matrices display notable differences in growth and behavior (Baker and Chen, 2012; Stock et al., 2016). Studying cell population dynamics without the environmental context may lead to inaccurate conclusions; computational models provide a method for exploring cell behavior within precisely controlled, dynamic environments.

Agent-based models (ABMs) are particularly well-suited for addressing these areas to explore how complex, heterogeneous interactions at the cellular level result in the emergence of spatial and temporal dynamics at the cell population level (Thorne et al., 2007; Yu and Bagheri, 2016). ABMs are a bottom-up modeling technique in which autonomous agents follow a set of rules that define their actions and interactions with each other and their environment (Bonabeau, 2002). Specifically, ABMs can readily incorporate agent heterogeneity and environmental dynamics with high precision and resolution. Classically used in the social sciences, ABMs have become increasingly popular for studying emergent behavior in biological systems, including bacterial biofilms and infection (Segovia-Juarez et al., 2004; Gorochowski et al., 2012), tumor growth (Enderling et al., 2009; Mehdizadeh et al., 2013; Walpole et al., 2015; Norton et al., 2017), and immune interactions (Folcik et al., 2007; Pienaar et al., 2015).

In this study, we introduce an extensible ABM framework designed to interrogate heterogeneous cell systems within dynamic environments with high spatial and temporal resolution. A key feature of the model is flexibility in defining agents and environments through interfaces and modular intracellular components. We use the presented ABM to investigate emergent dynamics in three relevant case studies: (i) to compare cell population dynamics between colony and tissue contexts, (ii) to explore competition between cell populations, and (iii) to investigate the impact of heterogeneity on clonal evolution and emergent dynamics.

## 2. Results

ARCADE (Agent-based Representation of Cells And Dynamic Environments) is built in Java, using the MASON library for multi-agent scheduling and simulation (Luke et al., 2005) along with a custom, extensible, interface-based framework for defining agents and environments. At the start of a simulation, selected agents and environments are added. MASON then runs the simulation by stepping through agent rules at each time step (representing 1 min, called ticks). A single simulation of 14 days (20,160 ticks) requires 5–10 min of CPU time on a computer with Intel^®^ Core i7 Processor (8x 3.40 GHz) and 19.5 GB of RAM.

### 2.1. Interfaces Provide an Extensible Modeling Approach

Java interfaces act as contracts between the underlying model framework and the implementing classes, guaranteeing that a certain set of methods are provided. By abstracting out how an agent interacts with its environment, the model is agnostic to a specific system and can be easily extended and customized.

**Figure 1:**
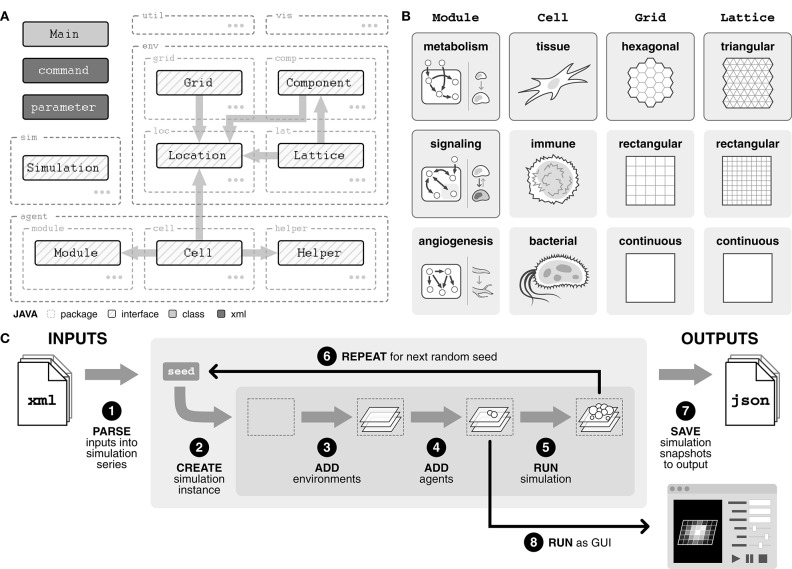
Overview of agent-based model framework. **(A)** Diagram of package structure and interfaces. Agents include Cell, Module, and Helper and environments include Grid, Lattice, Component, and Location. By importing an interface, a class is guaranteed a certain set of methods with which it can interact with objects of the imported interface. **(B)** Interfaces can be implemented into concrete classes in a variety of ways, depending on the system of interest. Classes with solid border are implemented in our model. **(C)** Overview of the modeling pipeline. Inputs, defined with an XML (.xml) file, are parsed to create a simulation series. Within the simulation series, for each random seed, a simulation instance is created. Environments and agents are added to the simulation instance. The simulation is stepped, and data is output to a JSON (.json) file. Alternatively, the simulation can be run in GUI mode.

Broadly, the model comprises three main packages—simulation (sim), agents (agent), and environments (env)—as well as visualization (vis) and utility (util) packages (Figure 1A). The simulation package handles the processing of inputs into simulation series, running the simulations, and saving simulation results to output.

There are three types of agents. First, Cell agents represent the physical cells within the system, such as tissue, immune, or bacterial cells (Figure 1B). These agents are introduced into the simulation and at each tick, they follow their rules defining how they interact with their surroundings. Second, Module agents are subcellular entities that represent a certain function or behavior within a cell, such as metabolism, signaling, and angiogenesis (Figure 1B). Finally, Helper agents provide a mechanism for (i) outside perturbations to the system, such as the introduction of new cell agents or a wound, and (ii) time delayed behaviors by Cell agents, such as division or movement.

The environment is divided into three distinct layers, all of which are integrated through a Location object. The Grid is an abstract layer on which cell agents are contained and can be defined in a variety of geometries (Figure 1B). Each Lattice layer tracks nutrients or molecules of interest, such as glucose or oxygen, and can also be defined in a variety of geometries (Figure 1B). The geometry of the Lattice layers does not necessarily need to match the geometry of the Grid, allowing flexibility in how the environment is defined. Finally, Component layers provide a mechanism for (i) changes in the Lattice layers, such as diffusion or introduction of a drug, and (ii) physical entities within the environment, such as a capillary bed or matrix scaffolding.

### 2.2. Modeling Pipeline Emphasizes Flexible Inputs and High-Resolution Outputs

The model can be run both in GUI form, for real-time visualization of the simulation, or directly through command line for rapid simulation (Figure 1C). Simulations are defined using an XML (.xml) file describing one or more simulation *series* (Supplementary Figure 1A). Simulations within a series only differ in random seed, analogous to experimental replicates, and multiple series can be defined within a single input file.

Each series is created by parsing the input file for three tags: (i) simulation, which specifies model size as well as any profilers for capturing simulation data, (ii) agents, which describes the composition and parameters of cell agent populations, and (iii) environment which defines environment parameters (Figure 1C, Supplementary Figure 1A). For each seed, a simulation instance is created. Environments and agents are added into the simulation instance, and then the simulation is run for the defined number of ticks. This process is repeated for all random seeds in the series. Alternatively, if the GUI version is selected, the simulation is run through the GUI interface once environments and agents have been added.

Simulation outputs are saved as JSON (.json) files, a common, lightweight file format that uses human-readable text to store data (Supplementary Figure 1B). Each output file includes a summary of the input file, full parameter lists for every cell population, and location and cell information for all cells at selected timepoints during the simulation.

### 2.3. Tissue Cell Implementation Exhibits Representative Growth Dynamics

**Figure 2:**
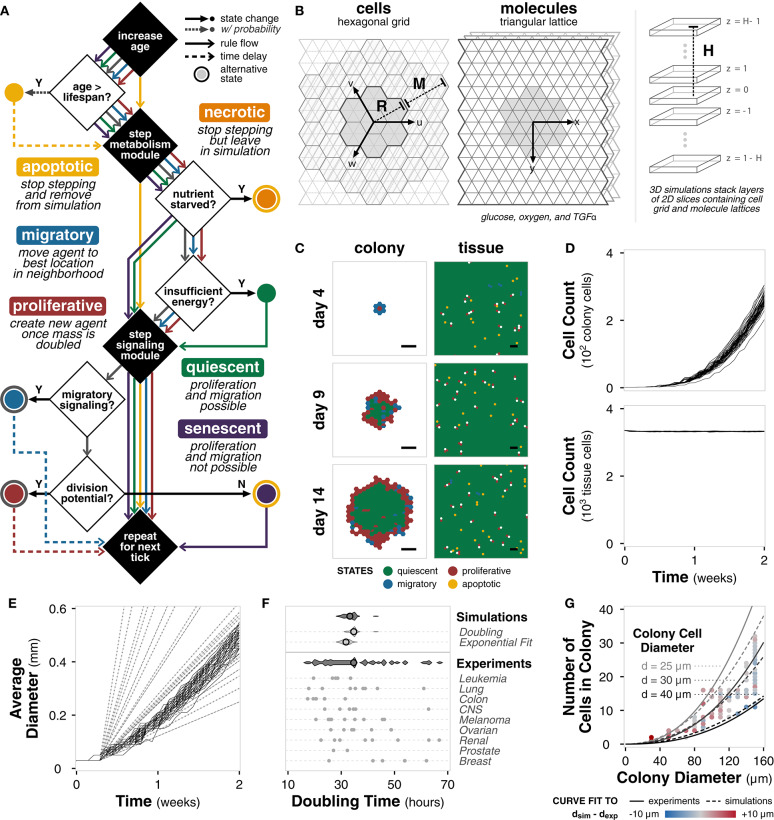
Tissue cell implementation. **(A)** Flowchart outlining tissue cell agent states and the rules governing transitions between them at each tick of the simulation. **(B)** Diagram of the simulation environment structure comprising a hexagonal grid for cells and triangular lattices for molecules. Environment size is defined by radius *R* from the center hexagon and a margin *M* between the cell grid and the molecule lattices. For 3D simulations, layers of 2D simulations are stacked at a height *H* from the center layer. **(C)** Spatial distribution of cell states for colony (left) and tissue (right) growth for a single example replicate (random seed 0) at different timepoints during the simulation. Scale bars represent 100 μm. **(D)** Plot of total cell count for colony (top) and tissue (bottom) growth for each of *n* = 50 replicate simulations of the model with default parameters and settings. Each line shows the trajectory for a single simulation. **(E)** Plot of average colony diameter for each of *n* = 50 replicate simulations of the model with default parameters and settings. Each line shows the trajectory for a single simulation. Dashed and dotted lines indicate experimentally observed diameters (Conger and Ziskin, 1983; Brú et al., 2003), respectively. **(F)** Violin plots of doubling times for the simulation (*n* = 50) calculated using (i) cell count doublings at *t* = 7 days and (ii) exponential curve fit to the first 7 days of growth compared to doubling times of the cancer cell lines in the NCI-60 panel (Alley et al., 1988), both aggregated and separated by pathology. Black circle indicates mean. **(G)** Scatter plot of colony diameter and number of cells in the colony for colonies less than 160 μm in diameter across *n* = 50 replicate simulations. Solid lines show the relationship between colony diameter, number of cells in the colony, and diameter of a colony cell using an equation fit to experimental data (Meyskens et al., 1984). Dotted lines show the same relationship for an equation of the same form fit to the simulation results. Colors indicate the difference between the cell diameter calculated directly from the simulation data and the cell diameter predicted by the experimental fit.

With the framework and pipeline in place, specific classes for tissue cell agents are implemented within a hexagonal and triangular environment (Supplementary Figure 2A). Each tissue cell agent contains a metabolism and signaling module (described in the following section) and can be in one of seven states: apoptotic, necrotic, quiescent, migratory, proliferative, senescent, and undecided (Methods). At each tick, each agent steps through specific decisions based on its current state (Figure 2A). Briefly, a cell agent increases in age and evaluates if its age is greater than the defined lifespan. If so, it becomes apoptotic. The metabolism module is simulated to update energy and volume of the cell. If the cell is nutrient starved, it becomes necrotic; if there is insufficient energy, it becomes quiescent. The signaling module is simulated to decide between migratory and proliferative states for undecided cells. Cells who have reached their division limit become senescent. This process repeats for all cell agents at the current tick, and then for each tick of the simulation. Default cell parameters are derived from literature (Supplementary Table 1). In addition, we develop a null model for comparison in which agents simulate their metabolism and signaling modules, but instead randomly select a cell state (Supplementary Figure 2B).

The environment comprises a hexagonal grid containing the cell agents and three triangular lattices in which glucose, oxygen, and a signaling molecule TGFα diffuse (Figure 2B). Each hexagon is 30 μm in diameter (side-to-side) and contains, on average, 2-3 cells depending on total cell volume. The grid is *R* = 34 hexagons in radius with an *M* = 6 hexagon margin, for a total environment diameter of approximately 2 mm, which is consistent with experimental observations of the limiting radius for non-vascularized tumors (Heymach et al., 2010). Because the hexagonal grid and triangular lattices explicitly account for volume, the 2D simulations are representative of a 3D cross section. Simulations in 3D (*H* > 1) utilize layers of these 2D simulations, with alternating cell grid offsets to prevent vertical cell stacking. Default environmental parameters are derived from literature (Supplementary Table 2).

With high temporal and spatial resolution, we monitor a number of features over the course of the simulation. We ran sample growth simulations of colony and tissue growth for 14 days with *n* = 50 replicates (i.e., different random seeds) and timepoints taken every 12 h with default, untuned parameters (Supplementary Table 3). The colony growth simulations are initialized with a single cell agent, whereas the tissue growth simulations are initialized with one agent in every location.

For a single simulation, we can capture the spatial distribution of cell states (Figure 2C). For colony growth, as observed experimentally, there is a rim of active cells—proliferative and migratory—surrounding the inactive, quiescent core (Freyer and Sutherland, 1986; Brú et al., 2003). The rim spans approximately 2−4 hexagonal locations (equivalent to 60−120 μm), consistent with literature measurements, which span 25−100 μm across a variety of glucose and oxygen concentrations (Freyer and Sutherland, 1986). For tissue growth, there exists tissue homeostasis with the majority of cells in a quiescent state. Neither the distribution of the cell states nor the thickness of the rim are specified in the model. Instead, these biologically relevant behaviors emerge directly from agent and environment interactions. In contrast, the null model, initialized with a single cell agent as well as multiple cell agents, fails to show the observed emergent spatial behavior (Supplementary Figure 2C). Instead, the cells begin with an equal distribution of all cell states and quickly fall into irreversible terminal states (necrotic, apoptotic, and senescent) whereas the full rule set maintains active cell states (Supplementary Figure 2D).

The total number of cells over time are shown in Figure 2D. Fitting an exponential curve to the number of colony cells for the first 7 days gives *r*^2^ = 0.98 ± 0.01 across the replicates, indicating clear early exponential growth. The number of cells in tissue growth quickly reaches a steady state, further indicating tissue homeostasis. The diameter growth rate of the colony cells is 1.45 ± 0.09 μm · h^−1^, which falls well within the experimentally reported range of 3.78 ± 3.14 μm · h^−1^ for 15 *in vitro* cell lines (Brú et al., 2003) and 1.89 ± 1.09 μm · h^−1^ for 8 *in vitro* tumor spheroids (Conger and Ziskin, 1983). The linear increase in diameter and early exponential increase in cell number, both experimentally observed behaviors (Brú et al., 2003; Talkington and Durrett, 2015), emerge without explicitly defining these growth dynamics in the model.

Finally, we consider emergent phenomena at the single cell level. The average doubling time of cells in the simulation, calculated at 7 days, is 34.6 ± 1.5 or 31.8 ± 1.4 h depending on calculation method, is well within literature values of doubling time for human cancer cell lines (Figure 2E) (Alley et al., 1988). We also find that relationship between colony diameter, cell number, and cell diameter match the literature reported relationship between these features for tumor populations (Figure 2F) (Meyskens et al., 1984). Again, we note that the model was never trained to meet these objectives; doubling time and the cell size relationships emerge *de novo*.

For the following case studies, we consider temporal, spatial, and parametric emergent phenomena quantified using three metrics: growth rate, symmetry, and cell cycle length, respectively (Methods). Note that cell cycle length is not equivalent to doubling time; cycle length is the amount of time a cell takes to complete its cell cycle and is tracked per cell while doubling time is calculated based on change in population cell counts between two timepoints. Model parameters are not specifically tuned or derived to provide these specific emergent outcomes. In addition, these metrics are not a function of initial state conditions, which allows us to compare results between simulations.

### 2.4. Module Complexity and Model Resolution Impact Emergent Population Dynamics

To determine how the complexity of subcellular modules, and thus model resolution, impacts emergent cell population dynamics, we introduce metabolism and signaling modules with complex, medium, simple, and random mechanistic detail. Here, we consider simulations for every combination of metabolism and signaling module. Note that for case study simulations, the complex metabolism and complex signaling modules are used.

**Figure 3:**
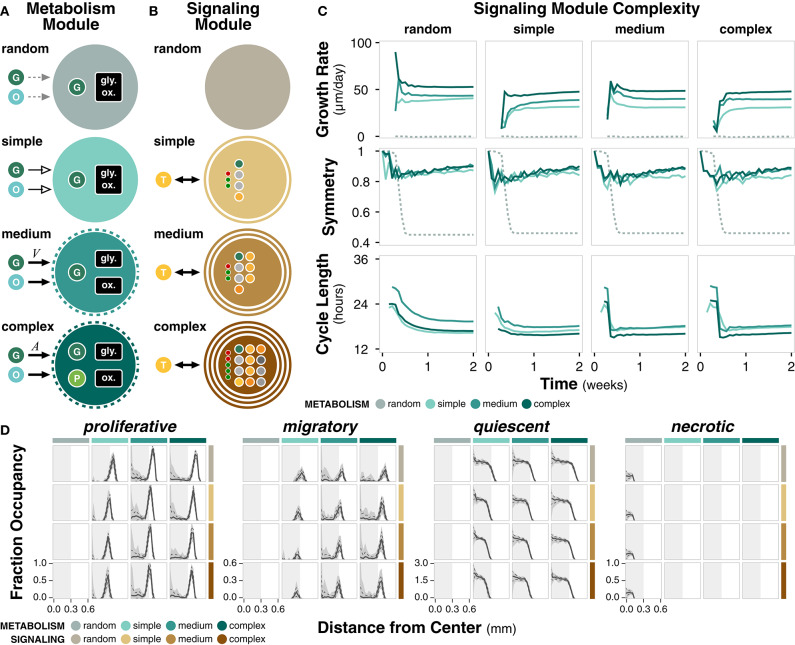
Metabolism and signaling module complexity. **(A)** Diagram summarizing the differences between complexities of the metabolism module. All modules uptake glucose (G) and oxygen (O) from the environment through various mechanisms. Cell size regulation by autophagy is indicated by dotted ring. Only complex metabolism explicitly accounts for a pyruvate (P) intermediate. Nutrient uptake can be variable (solid, black arrow), constant (solid, empty arrow), or random (dotted, gray arrow). **(B)** Diagram summarizing the differences between complexities of the signaling module. The non-random modules interact with extracellular TGFα (T). Number of rings indicate how many cellular compartments (i.e., membrane, cytoplasm, and/or nucleus) are explicitly included in the signaling network. Large dots denote molecular species within the network and small dots denote regulatory interactions. **(C)** Time course of growth rate, symmetry, and cycle length for different complexities of the metabolism and signaling modules, grouped by signaling module complexity. **(D)** Distribution of cell states as a function of distance from the center of the colony at *t* = 2 weeks. Solid line, dotted line, and shaded area denote the mean, standard deviation, and range across *n* = 20 replicates. Light gray rectangle is a visual reference for a distance of 0.3 mm from the center across all cell states.

The metabolism module governs changes in cell energy and volume as a function of external nutrient availability and internal cell state (Figure 3A; Methods). Complex metabolism explicitly accounts for both glycolysis and oxidative phosphorylation pathways and produces an internal pyruvate intermediate. Glucose uptake is based on cell surface area, which acts as a proxy for the number of glucose receptors. Medium metabolism implicitly accounts for glycolysis and oxidative phosphorylation and glucose uptake is based on cell volume. Both complex and medium metabolism use autophagy to regulate cell size. Simple metabolism assumes constant glucose uptake, energy production, and growth rate. Random metabolism takes up a random fraction of the external nutrients and uses a random fraction of internal glucose to produce cell mass. Metabolism module parameters are derived from literature (Supplementary Table 4).

The signaling module governs the decision between proliferative and migratory states as a function of the change in concentration of active PLCγ (Figure 3B; Methods). Complex signaling is a simplification of an established EGFR signaling network (Zhang et al., 2007) consisting of 12 species and five regulatory edges, spanning the nucleus, cytoplasm, and cell membrane. Medium signaling does not explicitly include the nuclear compartment, resulting in a network with seven species and three regulatory edges. Simple signaling further removes the cell membrane compartment for a network with four species and three regulatory edges. Random signaling is uncoupled to external TGFα and selects between the two states with a certain probability. Signaling module parameters are derived from literature (Supplementary Table 5).

We first consider the effect of these modules on the external concentrations of glucose, oxygen, and TGFα, independent of cell state and cell decision processes. Cell agents, fixed in a quiescent state with no rules, are introduced into the environment. When these agents contain only the metabolism module, both glucose and oxygen consumption decrease with increasing metabolism complexity (Supplementary Figure 3A, left). With random metabolism, glucose and oxygen consumption are similar to complex metabolism given the parameterization, suggesting that a correctly parameterized simplification may be sufficient if only external nutrient concentrations are of interest. There are no time dependent effects; glucose and oxygen consumption quickly reach a steady state.

However, when these fixed state agents contain only the signaling module, TGFα shows time dependent effects (Supplementary Figure 3A, right). Complex signaling exhibits an early spike in TGFα before returning to equilibrium whereas medium and simple signaling both exhibit a dip in TGFα and establish new equilibriums. The major difference between complex and simple/medium signaling modules is the number of regulatory edges, emphasizing the importance of regulation in biological systems. As expected, (i) TGFα is unaffected when agents contain only the metabolism module and (ii) glucose and oxygen are unaffected when agents contain only the signaling module.

For agents fixed in a quiescent state with pairwise combinations of modules, there are no significant differences compared to simulating the modules in isolation (Supplementary Figure 3B, left). When the full rule set is added to cell agents, glucose and oxygen consumption become more dependent on module complexities (Supplementary Figure 3B, right).

We ran simulations for every combination of metabolism and signaling module (Supplementary Table 3). Simulations reflect 14 days of growth (timepoints taken every 12 h) and reflect outcomes across 20 replicates. Growth rate increases with higher non-random metabolism complexity, suggesting more efficient utilization of nutrients to meet energetic and growth requirements (Figure 3C). For a given metabolism module complexity, signaling complexity changes early growth rate dynamics (Supplementary Figure 3C), perhaps due to an early compromise between the proliferation and migration governed by PLCγ. The random metabolism module is unable to meet energetic demands, resulting in negligible cell growth. Symmetry increases slightly with increasing metabolism complexity for a given signaling complexity or decreasing signaling complexity for a given metabolism complexity (Figure 3C; Supplementary Figure 3C). Overall, long term symmetry is unaffected by module complexity, except in simulations with random metabolism in which symmetry is significantly lower. Cell cycle length ranges between 16 and 24 h. Higher metabolism complexity generally results in shorter cell cycles; cells are able to more effectively utilize nutrients to produce cell mass necessary for division (Figure 3C; Supplementary Figure 3C). Within a given metabolism module, higher complexity signaling results in a slightly shorter early cell cycle (Supplementary Figure 3C).

All combinations of modules except those with random metabolism produce cell colonies with a quiescent core surrounded by a proliferative and migratory rim (Figure 3D). There is a distribution of apoptotic cells for all cases except for random metabolism, which results in a necrotic core (Figure 3D; Supplementary Figure 3D). This difference further highlights that the random metabolism module is unable to regulate nutrient usage to produce sufficient energy for the cell.

Overall, we observe key spatial and temporal behaviors that only occur at certain levels of module complexity. For example, extracellular TGFα concentration profiles are highly dependent on the complexity of the signaling module and a necrotic core emerges without a minimal complexity of the metabolism module. Identifying such relationships offer guidelines on the resolution of a computational model necessary to capture specific behaviors in a given biological system.

### 2.5. Case Study 1: Cell Population Dynamics Differ Between Colony and Tissue Contexts

**Figure 4:**
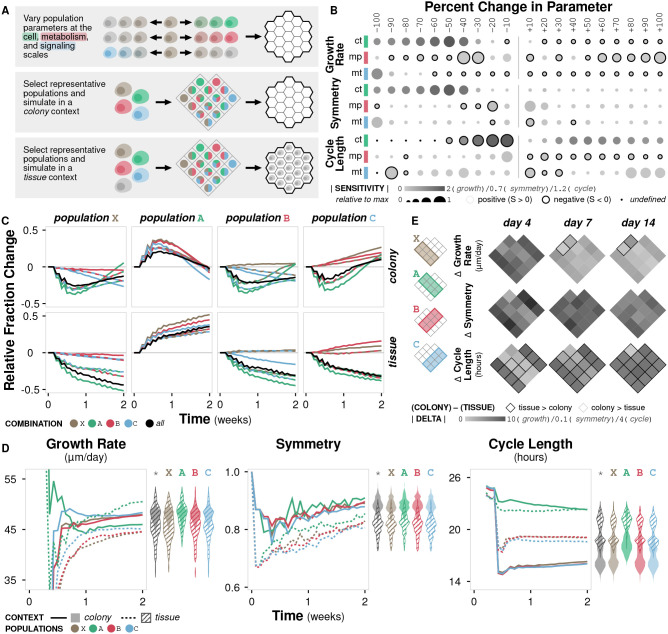
Case study 1: Context. **(A)** Diagram of the three sets of simulations. First, three parameters at the cell, metabolism, and signaling scales (crowding tolerance, metabolic preference, and migratory threshold, respectively) were varied +/− 100% (increments of 10%) and initialized onto an empty environment. Second, combinations of representative cell populations (A, B, C, and X) were initialized onto an empty environment to represent a colony context. Third, combinations of representative cell populations (A, B, C, and X) were initialized onto an environment containing a generic cell population to represent a tissue context. **(B)** Sensitivity of three metrics to variation in the three parameters calculated as (*y* − *y*_0_)*x*_0_/(*x* − *x*_0_)*y*_0_ where *y* is the metric value and *x* is the parameter value. Circle size indicates relative fold change in sensitivity to the maximum for a given metric and parameter, circle color indicates absolute sensitivity, and inverse relationships are indicated by a black border. **(C)** Relative change in population fraction for each of the four representative populations over time across all combinations under colony and tissue contexts. Color indicates the other populations included in the simulation; black indicates all three other populations where included. **(D)** Time course of metric values for the four representative populations under colony and tissue contexts. Violin plots show distribution of the metric value between contexts at time *t* = 2 weeks for all population combinations (^*^) or for population combinations including the indicated population. **(E)** Heat map of the change in metric value between the (colony) − (tissue) contexts at different timepoints for all population combinations.

*In vitro* studies are ubiquitous in biological research, but they remain limited in their ability to replicate the rich context of the microenvironment (Kim et al., 2004; Hickman et al., 2014). This limitation can result in misleading conclusions that are not relevant or consistent *in vivo* (Fràter-Schröder et al., 1987; Toledo and Wahl, 2006) or even in three-dimensional *in vitro* culture (Wang et al., 1998). Our model can be used to identify differences in emergent behavior as a function of context. In doing so, we are able to (i) distinguish between cases where the difference is irrelevant or negligible and assume observations made *in vitro* hold *in vivo*, and vice versa, as well as (ii) guide experimental design to avoid or compensate in cases where the difference is significant. Here, we simulate cells with variations in three parameters (crowding tolerance, metabolic preference, and migratory threshold) in both colony and tissue contexts, representing *in vitro* and *in vivo* experiments, respectively (Figure 4A; Methods).

Growth rate is non-linearly sensitive to changes in crowding tolerance and somewhat linearly sensitive to changes in metabolic preference and migratory threshold (Figure 4B; Supplementary Figure 4A). Large decreases in crowding tolerance (< −40%) leads to a significant drop in growth rate as cells are unable to successfully divide due to physical constraints. No migratory threshold (−100%) also results in a drop in growth rate as cells are unable to become proliferative. Symmetry diminishes with both decreased crowding tolerance and migratory threshold, but is essentially unaffected by metabolic preference (Supplementary Figure 4A). Cell cycle length is sensitive to crowding tolerance, and, to a lesser degree, migratory threshold (Supplementary Figure 4A). Overall, the sensitivities of different metrics to changes in parameter values are variable, with crowding tolerance exhibiting highly non-linear trends.

We define the three representative cell populations based on known cancerous phenotypes: A (crowding tolerance at +50% of baseline), B (metabolic preference at +50% of baseline), and C (migratory threshold at −50% of baseline). We also define an unmodified cell population X (all parameters at baseline). Each population exhibits distinct trends in population fraction over time when simulated in combinations. The relative fraction of population X generally decreases, confirming that all the modified populations (A, B, and C) have growth advantages over the unmodified population (Figure 4C). The relative fraction of population A generally increases (Figure 4C). Populations B and C show variable changes in fraction depending on which other populations are present; they are able to outgrow population X but not population A, and population C is able to outgrow population B (Figure 4C). These colony trends for populations X, B, and C hold in the tissue context, but the early increase then gradual return to the initial fraction for population A seen in the colony context is not observed in the tissue context (Figure 4C).

With the addition of the generic background cell population in the tissue simulations, increased tolerance for crowding becomes a more valuable phenotype, resulting in a growth rate comparable to that in the colony simulations (Figure 4D). In the colony context, the advantage of increased crowding tolerance (population A) becomes less important after the initial burst of growth (Figure 4D). In the tissue context, there is significantly lower symmetry for all populations (A, B, C, and X) and higher cycle times for all populations, except A (Figure 4D). While symmetry and cell cycle length show clear separation in trajectories between the colony and tissue contexts, growth rate exhibits overlap between contexts, suggesting that growth rate is less sensitive overall to the addition of a generic background population.

In general, when simulating combinations of the four representative populations in a colony context, the resulting overall population symmetry and cycle length are near the average of the constituent populations (Supplementary Figure 4B). However, growth rate tends to be higher than the average of the constituent populations when population A is included, even though population A alone has the lowest growth rate. This behavior suggests a synergy in cases where population A is grown with other populations. In the tissue context, population growth rate and symmetry are near the average of the constituent populations, but cycle length is more likely to favor one of the constituent populations, demonstrating that the addition of a generic background population changes the emergent dynamics of the system such that certain phenotypic modifications become more or less advantageous (Supplementary Figure 4B).

Growth rate is generally higher in colony contexts, though the increase depends on the constituent populations and decreases over time (Figure 4E). Symmetry is consistently higher in colony contexts. Cell cycle length is higher in tissue contexts, except for combinations containing population A, where cycle length is essentially equal between the two contexts during early growth (Figure 4E).

Overall, we observe significant differences between the colony and tissue simulation contexts across all three metrics of emergent phenomena. The tissue context simulations generally exhibit lower growth rates, decreased symmetry, and higher cell cycle lengths, though population-dependent effects do exist. These differences might help explain observations in cell culture that are not consistent in animal models and highlights the importance of context when designing both computational and experimental models of biological systems.

### 2.6. Case Study 2: Cell and Module Parameters Govern Competitive Fitness Between Cell Populations

**Figure 5:**
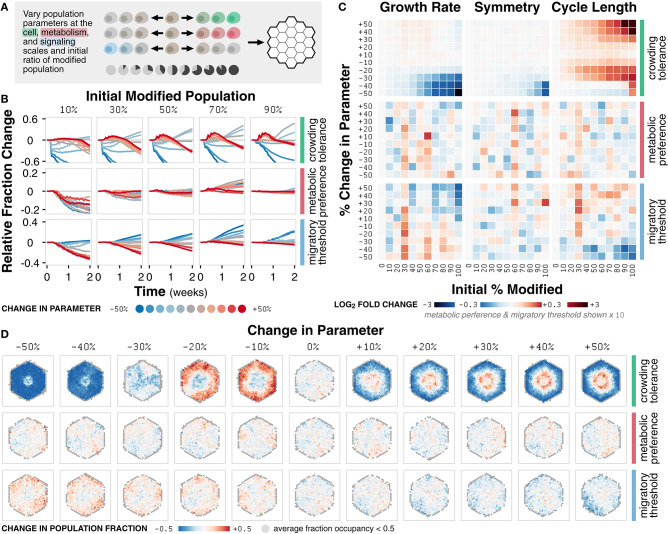
Case study 2: Competition. **(A)** Diagram of the set of simulations. Three parameters at the cell, metabolism, and signaling scales (crowding tolerance, metabolic preference, and migratory threshold, respectively) were varied +/− 50% (increments of 10%) and initialized in ratios of 0 to 100% (increments of 10%) with a basal, unmodified cell population onto an empty environment. **(B)** Relative change in population fraction for the modified population over time for different changes in parameter at selected initial ratios of the modified population. **(C)** Heat maps of fold change in metric value across *n* = 20 replicates for different changes in parameter and initial ratios relative to a 0% change in parameter at *t* = 2 weeks. **(D)** Spatial distribution of change in fraction of the modified population at *t* = 2 weeks across *n* = 20 replicates for simulations initialized with an equal mixture of the modified and basal cell populations. Locations with less than 0.5 fraction occupancy across replicates are shown in gray.

Biological systems rarely contain only a single population of cells; they comprise complex cell-cell interactions that drive emergent dynamics of the system. Cellular competition has been shown to impact dynamics in a variety of contexts including development, aging, and cancer (Gregorio et al., 2016; Merino et al., 2016). Co-culture systems have been used to study such phenomena (Kirkpatrick et al., 2011; Goers et al., 2014). Several variables must be considered—cell composition, relative seeding and spatial separation, culture dimensionality and local environment—all of which affect temporal and spatial observations and present challenges for data acquisition (Kirkpatrick et al., 2011; Goers et al., 2014). Our model provides a platform for *in silico* co-culture in which these variables can be easily and precisely tuned and controlled. Here, we simulate a modified population along with an unmodified, basal population to specifically interrogate the how differences in cell phenotype and relative seeding affect competitive fitness (Figure 5A; Methods).

The crowding tolerance parameter significantly impacts the fraction and dominance of the modified population in co-culture simulations (Figure 5B). Significant decreases in crowding tolerance (−30, −40, and −50%) lead to a decrease in the fraction of modified population relative to the initial fraction. Any increase (+10, +20, +30, +40, and +50%) or, unexpectedly, slight negative decrease (−10% and −20%) to crowding tolerance leads to an increase in the fraction of the modified population.

Changes in the metabolic preference parameter result in non-linear changes in the fraction of the modified population (Figure 5B). Modifying the migratory threshold parameter follows a linear trend; an increase or decrease in parameter values results in a decrease or increase in the fraction of modified population, respectively (Figure 5B). The non-linear trend of metabolic preference indicates that the fraction of energy derived from glycolysis has a complex relationship to population fitness whereas the linear trend of migratory threshold suggests that a cell more likely to commit to migration instead of proliferation is a more competitive phenotype relative to the basal population. For crowding tolerance, the multimodal responses indicate that both an increased and decreased (to a certain limit) tolerance to crowding can be advantageous.

The trends observed in changes in modified population fraction as a function of modified parameter are reflected in emergent behavior (Figure 5C). Differences in growth rate due to changes in crowding tolerance are more prominent for higher initial modified population (Supplementary Figure 5A). Variations in the crowding tolerance parameter represent different tolerances to mechanical stress during the competition for space (Merino et al., 2016). The modified population with an increased crowding tolerance is able to pack more densely in the core of the cell colony whereas the population with a slightly decreased tolerance is incentivized to grow outward; both strategies are sufficient to outcompete the basal population (Figure 5D; Supplementary Figure 5B).

Symmetry, a function of spatial distribution, is mostly unaffected by competition, except with a decrease due to decrease in crowding tolerance at high initial modified population (Figure 5C; Supplementary Figure 5A). Cell colonies that look spatially similar may have distinctly different composition at the subcellular level (Supplementary Figure 5C). For example, tumors may appear spatially homogeneous despite being composed of highly diverse subpopulations; a biopsy may only represent a small fraction this diversity (Poleszczuk et al., 2015). Similarly, microbial colonies, which are largely indistinguishable spatially, may contain highly diverse mixtures of the component cells in which competition is driven by cell morphology (Smith et al., 2016).

Cell cycle length is also essentially unaffected for metabolic preference and migratory threshold (Figure 5C). However, increased and slightly decreased crowding tolerance leads to increased cell cycle length (Figure 5C; Supplementary Figure 5A). The increased tolerance for crowding results in greater competition for nutrients, requiring more time for cell growth before division. However, the benefit of increased tolerance for mechanical stress outweighs the disadvantage of a slower cell cycle; this tradeoff allows the modified population to outcompete the basal population and highlights the relative (and arguably non-intuitive) contributions of different modes of competition.

Overall, we observe both linear (migratory threshold), non-linear (metabolic preference) and multimodal (crowding tolerance) relationships between the parameter values of the modified population and the emergent behavior of the system. The high temporal and spatial resolution of our simulations, in combination with parametric sensitivity analysis, help identify when, where, and how the modified population is able to outcompete the basal population. In addition, identifying the linearity and transition points of these relationships provide insight into the mechanisms of the underlying cell-cell interactions. The multimodal relationship between modifications in crowding tolerance and growth dynamics, for example, demonstrates that there exist two separate mechanisms by which cells with an increased or decreased tolerance for mechanical stress can successfully outcompete another population.

### 2.7. Case Study 3: Intra- and Intercellular Heterogeneity Impact Clonal Evolution and Emergent Dynamics

**Figure 6:**
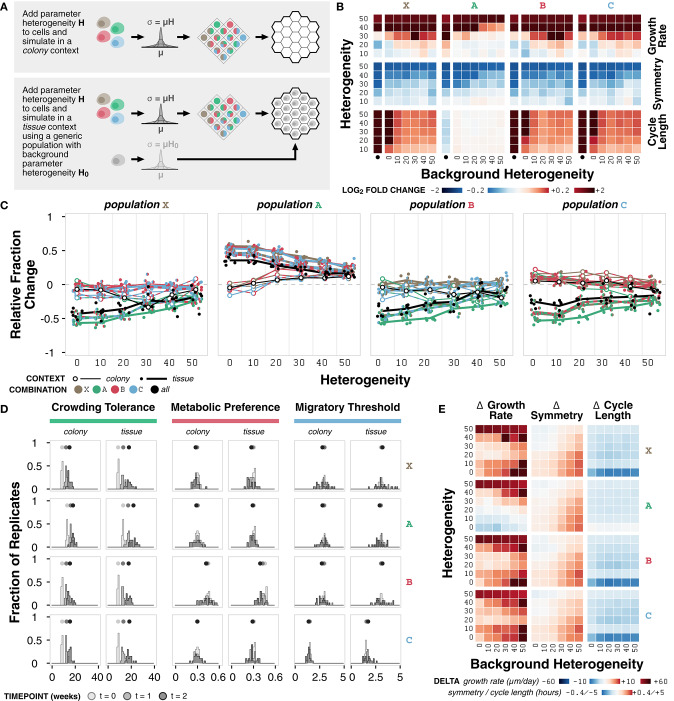
Case study 3: Heterogeneity. **(A)** Diagram of the two sets of simulations. First, combinations of representative cell populations (A, B, C, and X) with heterogeneity *H* varied at 0, 10, 20, 30, 40, and 50% were initialized onto an empty environment to represent a colony context. Second, combinations of representative cell populations (A, B, C, and X) with heterogeneity *H* varied at 0, 10, 20, 30, 40, and 50% were initialized onto an environment containing a generic cell population with background heterogeneity *H*_0_ varied at 0, 10, 20, 30, 40, and 50% to represent a tissue context. **(B)** Heat maps of fold change in metrics for each representative cell population across different amounts of heterogeneity relative to the non-heterogeneous (*H* = 0) case at *t* = 2 weeks. The colony context (no background population) is indicated by the bullet (•). **(C)** Relative change in population fraction for each of the four representative populations for all combinations as a function of heterogeneity at *t* = 2 weeks. Color indicates the other populations included in the simulation; black indicates all three other populations where included. Lines connect average values for the colony context (thin) and averages across values for different background heterogeneities for the tissue context (thick) for each combination. **(D)** Distributions parameter means across *n* = 20 replicates at different timepoints under colony and tissue contexts for representative cell populations with *H* = *H*_0_ = 40. **(E)** Heat maps of change in metric value between the (colony with heterogeneity) – (tissue with heterogeneity) contexts for representative cell populations at *t* = 2 weeks.

Cell heterogeneity is an intrinsic property of biological systems, even within clonal populations (Raser, 2004; Lidstrom and Konopka, 2010; Marusyk et al., 2012). Advancements in experimental approaches have enabled observation and quantification of heterogeneity at the single cell level (Schmid et al., 2010; Walling and Shepard, 2011). However, while heterogeneity can be measured, it cannot be systematically varied. Given the ubiquitous nature of heterogeneity, it remains important to distinguish between functional variation that selectively arises to improve evolutionary fitness from intrinsic variation that arises from random fluctuations (Altschuler and Wu, 2010). Our model allows for explicit control of differences between cell populations, variation in cell parameters, and probabilities of stochastic processes. Here, we simulate growth in both colony and tissue contexts to explore how heterogeneity within and between cell populations impacts emergent responses (Figure 6A; Methods).

Growth rate increases with increasing heterogeneity in colony and tissue simulations (Figure 6B; Supplementary Figure 6A). Higher background heterogeneity corresponds to lower growth rate for all populations (A, B, C, and X) (Supplementary Figure 6B). Symmetry generally decreases for any increase in heterogeneity or background heterogeneity (Figure 6B; Supplementary Figures 6A,B). Interestingly, the change in growth rate and symmetry as heterogeneity increases is not consistent across different background heterogeneities, which suggests that background heterogeneity can mask the effects of heterogeneity in the population of interest (Figure 6B). Cell cycle length increases with increasing heterogeneity for most populations; population A is minimally affected (Figure 6B; Supplementary Figure 6A). Background heterogeneity does not have a clear relationship to cycle length; this emergent behavior appears to be less context-dependent and more population-dependent, as previously noted (Supplementary Figure 6B).

In general, regardless of context, population A increases and population X decreases in fraction when simulated in combination with other populations (Figure 6C). Population A persists best in colony contexts at higher heterogeneity (H ≥ 20) and persists best in tissue contexts at lower heterogeneity (*H* < 20) (Figure 6C). Change in population A fraction is unaffected by background heterogeneity (Supplementary Figure 6C). Populations X, B, and C do not have clear background heterogeneity trends, but generally exhibit better population fraction outcomes in tissue contexts as heterogeneity increases (Figure 6C).

The crowding tolerance parameter (Supplementary Table 1), which is already higher in population A, is one of the internal cell parameters now subject to heterogeneity in all four representative populations. The increased heterogeneity in the other populations, which are normally less competitive in tissue contexts than population A, provides a mechanism by which they can select for cells with a higher crowding tolerance. This hypothesis is further supported by the observation that populations B and C persist better in colony contexts, where there is a weaker selective pressure for higher crowding tolerance (Figure 6C). In addition, the distribution of the average value of the crowding tolerance parameter across the replicates shows a clear evolution toward a higher value (Figure 6D; Supplementary Figure 6D).

The metabolic preference parameter (Supplementary Table 4) shows a minor evolution toward a lower value in the tissue context for population B, in which the parameter was increased (Figure 6D). There is minimal evolution of the metabolic preference parameter in the other cell populations, suggesting that the basal value of metabolic preference was optimal for the given environment conditions in these simulations and that there exists a stronger selective pressure in the tissue context (Supplementary Figure 6D). The migratory threshold parameter (Supplementary Table 5) shows a minor increase toward a larger value at very high heterogeneity (Supplementary Figure 6D) for all populations. In almost all cases, the variance of the distribution in average parameter value across replicates increases from the initial distribution (Supplementary Figure 6E).

The changes in metrics between simulated colony and tissue contexts with the addition of heterogeneity generally match trends seen without heterogeneity for symmetry and cycle length: symmetry is higher and cycle length is lower in the colony context (Figure 6E). Growth rate shows a significant dependence on the degree of both heterogeneity and background heterogeneity. There exist critical values of heterogeneity and background heterogeneity for which growth rate in colony and tissue contexts are comparable (Figure 6E). In this system, background heterogeneity matters when growth rate and symmetry are of interest, but is less important for cycle length (Altschuler and Wu, 2010).

Overall, we observe heterogeneity-dependent emergent behavior in both colony and tissue contexts. Higher heterogeneity generally corresponds to higher growth rate, lower symmetry, and longer cycle lengths, though there are population-dependent effects as well. As a consequence of heterogeneity, cell populations evolve toward certain parameter values such as higher crowding tolerance. Exploring such trends identify how heterogeneity within and between populations shapes emergent population dynamics.

## 3. Discussion

Computational models are critical for understanding biological systems (Brodland, 2015; Yu and Bagheri, 2016). Agent-based modeling in particular has seen increasing applications in biology (An et al., 2009; Gorochowski, 2016). A number of agent-based modeling platforms exist, including Chaste (Mirams et al., 2013), CompuCell3D (Swat et al., 2012), and FLAME (Holcombe et al., 2012). We develop the first ABM that uses interfaces custom-built to formalize the interactions within and among cells and their environment.

We implemented a tissue cell system within the framework and demonstrate that, with literature-derived parameters and no additional parameter fitting, we produce biologically realistic growth dynamics that are agnostic to a specific cell population. Three case studies investigating cellular context, competition, and heterogeneity demonstrate how our model provides unique insight into biological systems in a manner that is infeasible to probe experimentally.

First, we analyze the impact of specific cell parameters and simulate representative populations in colony and tissue contexts. Second, we systematically vary cell population parameters and initial conditions of simulated co-culture experiments to evaluate cellular competition. Finally, we introduce tunable cell heterogeneity, both within the representative populations and between the representative and background populations. Tracking temporal, spatial, and single-cell data of each simulation across multiple replicates identifies non-linear trends and non-intuitive relationships. These observations offer hypotheses on the underlying mechanisms that could be validated experimentally.

Our framework is readily extensible across many biological systems, with applications in a variety of areas including drug development, personalized medicine, and synthetic biology. The model can be tuned to a specific disease or patient population context by varying cell parameters and altering the simulation environment. For example, we could simulate a highly glycolytic cancer growing in a patient with diabetes by increasing the metabolic preference for glycolysis parameter and setting a higher basal concentration of glucose in the simulation environment. We can then test how various perturbations, such as excision combined with radiation compared to excision alone, affects the growth of the tumor. Here, the model acts as an *testbed* with which to interrogate new strategies for drug design and treatment.

This framework can also catalyze a new approach to translational and personalized therapy by matching the model to biopsy and imaging data from a patient. Here, the model acts as a *proxy* with which to rapidly, inexpensively, and safely simulate a wide variety of possible interventions to develop patient-specific treatment regimes that offer more successful outcomes.

Finally, with the advent of engineered cell therapy (Kitada et al., 2018), this framework can uniquely redirect efforts in synthetic biology by predicting emergent outcomes. New agents, representing engineered immune cells with modules specific to their method of action, can be introduced to the model. Varying parameters and rules of the these agents, such as receptor density, binding strength, or target specificity, and observing the emergent response of the system can identify key design targets for effective cell therapy. Here, the model acts as a *tool* with which to predict novel system response in order to generate experimentally testable hypotheses.

In conclusion, our framework offers a new computational approach to interrogate the complexity and emergence of cell populations *de novo*. The intuitive nature of ABMs, in which rules can be explained with natural language and parameters are derived from literature values, helps bridge the gap between computational theory and experimental application and provides an opportunity for interdisciplinary collaboration (Cvijovic et al., 2014). We do not present a “whole cell” model nor seek to diminish the utility of reactive, equation-based approaches. Rather, we acknowledge the inherently multi-scale nature of biology and have designed a proactive, rule-based modeling framework to encourage the development of constituent parts by experts, and the investigation of their impact on emergent behavior in a variety of systems. This framework can serve as an invaluable resource that disrupts the status quo of current research efforts.

## 4. Methods

All source code for ARCADE is available on GitHub at https://github.com/bagherilab/ARCADE. MASON, a multi-agent simulation library required by the model, is available at https://cs.gmu.edu/~eclab/projects/mason/.

### 4.1. Model Agents

For the tissue cell implementation, seven cell states were defined: quiescent, migratory, proliferative, apoptotic, necrotic, senescent, and undecided. The state defines which rules the agent follows at each timepoint (Figure 2A). The undecided state acts as a transition state; undecided cells decide between migratory and proliferative states based on active PLCγ and the migratory threshold (MIGRA_THRESHOLD) through the signaling module (Zhang et al., 2007). Each cell agent is initialized with a volume drawn from a normal distribution (mean = CELL_VOL_AVG, standard deviation = CELL_VOL_RANGE) and an age drawn from a uniform distribution (between 0 and DEATH_AGE_RANGE).

#### 4.1.1. Quiescent

Cells can enter quiescence through a variety of mechanisms (Valcourt et al., 2012; Yao, 2014). Proliferating cells might become quiescent without completing the cell cycle due to contact inhibition (Gos et al., 2005), which occurs when (i) there are no neighboring locations into which it can divide or (ii) cell size exceeds the available space. Migratory cells might also become quiescent through contact inhibition (Abercrombie and Heaysman, 1953). Cells unable to meet energetic requirements become quiescent (Valcourt et al., 2012). Tissue cell agents exit quiescence through external growth signals, such as apoptosis of a neighboring cell inducing compensatory proliferation or the removal of contact inhibition (Valcourt et al., 2012; Yao, 2014).

#### 4.1.2. Migratory

Cells that decide to migrate create a helper agent that is called after a time delay corresponding to the distance the cells needs to move (HEX_SIZE) and its movement speed (MIGRA_RATE). The cell identifies all neighboring locations, including its current location, meeting the following criteria: (i) adding the new agent does not increase the total cell volume over the volume of the location (HEX_VOLUME), (ii) each agent, with the addition, exists at a height lower than their tolerable height (MAX_HEIGHT), and (iii) there are no more than 6 agents in the new location. To enforce normal cell density, no more than one healthy (H) cell agent is allowed in a location; the cancerous (C) and cancer stem cell (S) subtypes do not follow this additional constraint. To represent chemotactic movement (Zhang et al., 2007), each location *i* is assigned a score S based on glucose concentration $G_{i}$:

$$\begin{array}{l} S=\alpha \frac{R-r_{i}+1}{2}+\left(1-\alpha \right)\left[\beta \frac{G_{i}}{G^{{}^{\circ}}}+\left(1-\beta \right)u\right] \end{array}$$

where α is affinity (AFFINITY), *R* is the distance from the center of the migrating cell, *r* is the radial distance of location *i* from the center of the environment, β is accuracy (ACCURACY), $G^{{}^{\circ}}$ is the source concentration of glucose (CONC_GLUC), and *u* is a random number drawn from a uniform distribution *U*([0, 1]). If there are no locations that meet the criteria, the cell becomes quiescent, representing contact inhibition (Abercrombie and Heaysman, 1953).

#### 4.1.3. Proliferative

Cells that decide to proliferate create a helper agent that is stepped along with the rest of the agents until proliferation is complete or the cell is no longer able to proliferate. At each tick, the helper agent checks if (i) the cell is no longer proliferative, (ii) the cell no longer exists at a tolerable height, or (iii) there are no locations into which the cell can divide. For the latter two, the cell becomes quiescent, representing contact inhibition (Gos et al., 2005). Once (i) the cell has doubled in size, which is controlled by the metabolism module, and (ii) sufficient time for DNA synthesis has passed (SYNTHESIS_TIME), the helper creates a new cell agent by dividing the parent cell volume and module contents by 50%±5%. The division count for both cells is then incremented.

#### 4.1.4. Apoptotic

Cells that reach an age above the average life span (DEATH_AGE_AVG) have an increasingly high probability of undergoing apoptosis (Elmore, 2007), defined by a cumulative normal distribution (mean = DEATH_AGE_AVG, standard deviation = DEATH_AGE_RANGE). Cells that become apoptotic create a helper that is called after a time delay corresponding to the duration of apoptosis (DEATH_TIME). The helper removes the cell from the schedule and the grid—it is no longer stepped and it no longer occupies space in the environment—which represents the removal of cell debris and regulated nature of apoptosis (Edinger and Thompson, 2004). Compensatory proliferation is also mediated by the helper, which selects a quiescent neighbor of the cell and sets it to proliferate (Fan and Bergmann, 2008; Ryoo and Bergmann, 2012).

#### 4.1.5. Necrotic

Cells under sustained energy deficits (ENERGY_THRESHOLD) undergo necrosis (Edinger and Thompson, 2004; Zong, 2006). These cells also have a probability of undergoing apoptosis instead (NECRO_FRAC), to reflect the more continuous nature of the decision between, and morphology of, necrosis and apoptosis (Zong, 2006). Necrotic cells are removed from the schedule but remain in the grid—it is no longer stepped, but continues to occupy space—which represents the more disorganized nature of necrosis (Edinger and Thompson, 2004).

#### 4.1.6. Senescent

Cells that reach a replicative limit (DIVISION_POTENTIAL) have a probability (SENES_FRAC) of becoming senescent or apoptotic, due to uncertainty about what drives the decision between the two states (Childs et al., 2014). Senescent cells remain on the schedule and in the simulation, but are no longer able to proliferate (Campisi and d'Adda di Fagagna, 2007). Senescent cells might later become apoptotic/necrotic due to nutrient deficiency (Wang et al., 2016), but will not apoptose due to age (Campisi and d'Adda di Fagagna, 2007).

### 4.2. Model Environment

#### 4.2.1. Coupled Hexagonal and Triangular Grids

Cell agents exist on a hexagonal grid of radius *R* using a hexagonal coordinate system (*u, v, w, z*) such that (0, 0, 0, 0) is the center of the environment (Figure 2B). For three dimensional models (height *H* > 1), each hexagonal grid layer (*z* = 1 − *H*, ..., *H* − 1) has alternating offsets: offset *a* in the (−*u*, +*w*) and offset *b* in the (+*u*, −*v*) direction. The offsets prevent the cell agents from stacking in columns when simulated in 3D. Layer *z* = 0 always has no offset, offset *a* always has offset *b* above, and offset *b* always has no offset above. Given a location with coordinates (*u, v, w, z*), there are six equidistant neighboring locations in the same layer: (0,+1,-1,0), (0,-1,+1,0), (-1,+1,0,0), (+1,-1,0,0), (-1,0,+1,0), (+1,0,-1,0); three equidistant locations above: (0,0,0,+1), [(+1,0,-1,+1), (-1,+1,0,+1), (0,-1,+1,+1)], [(0,+1,-1,+1), (-1,0,+1,+1), (+1,-1,0,+1)]; and three equidistant locations below: (0,0,0,-1), [(-1,+1,0,-1), (0,-1,+1,-1), (+1,0,-1,-1)], [(0,+1,-1,-1), (-1,0,+1,-1), (+1,-1,0,-1)] where brackets indicate the offset of the layer: [no offset, offset *a*, and offset *b*].

Each molecule (oxygen, glucose, and TGFα) diffuses on triangular lattices using rectangular coordinate system (*x, y, z*) associated with the main hexagonal grid (Figure 2B). Glucose and oxygen are introduced from constant sources (CONC_GLUC and CONC_OXY). Each hexagonal location corresponds to six triangular lattice locations, indexed clockwise from the upper center triangle by position *p*. When cell agents interact with their local environment, average concentration across the six triangular locations is used.

#### 4.2.2. Molecule Diffusion

Diffusion of each molecule is calculated using a reaction-diffusion equation:

$$\begin{array}{l} \frac{\partial C}{\partial t}=D\nabla^{2}C+R_{a}+R_{s} \end{array}$$

where *C* is the concentration, D is diffusivity of the molecule in the environment (DIFF_GLUC, DIFF_OXY, or DIFF_TGF), *R*_*a*_ is the rate of consumption/production of the molecule by the cell agents, and *R*_*s*_ is the rate of production by the vasculature sources. Consumption and production of molecules (*R*_*a*_) and the source production (*R*_*s*_) are separately managed by cell agents and a sites component, respectively, which leaves:

$$\begin{array}{l} \frac{\partial C}{\partial t}=D\nabla^{2}C \end{array}$$

A finite difference approximation for this equation in triangular geometry (Huiskamp, 1991) is solved at each tick *t* to update the lattice concentrations for the next tick *t* + Δ*t*:

$$\begin{array}{l} C^{t+\Delta t}=C^{t}+\frac{4D\Delta t}{3\Delta s^{2}}\left(\overset{3}{\underset{i=1}{\sum }}C_{i}^{t}-3C^{t}\right)+\delta \frac{2D\Delta t}{\Delta z^{2}}\left(\overset{2}{\underset{j=1}{\sum }}C_{j}^{t}-2C^{t}\right) \end{array}$$

where Δ*t* is the time step (1 s), Δ*s* is the distance between two adjacent triangular locations (half of HEX_SIZE), Δ*z* is the distance between layers (MAX_HEIGHT), *i* indexes across the three triangular neighbors in a layer, *j* indexes across the two neighbors above and below the layer, and δ is 0 if *H* = 1 and 1 otherwise.

To check stability of the finite difference approximation, we perform a von Neumann stability analysis:

$$\begin{array}{l} \lambda =4D\Delta t\left(\frac{1}{\Delta s^{2}}+\delta \frac{1}{\Delta z^{2}}\right) \end{array}$$

For stability, 0 ≤ λ < 1. If not satisfied, we use a pseudo-steady state approximation:

$$\begin{array}{l} C^{t+\Delta t}=\frac{1}{3+\delta \frac{3\Delta s^{2}}{\Delta z^{2}}}\left[\overset{3}{\underset{i=1}{\sum }}C_{i}^{t}+\delta \frac{3\Delta s^{2}}{2\Delta z^{2}}\overset{2}{\underset{j=1}{\sum }}C_{j}^{t}\right] \end{array}$$

### 4.3. Metabolism Modules

All metabolism modules except for random metabolism account for glycolysis and oxidative phosphorylation pathways for producing energy (ATP) from glucose and oxygen with a metabolic preference μ for glycolysis over oxidative phosphorylation (META_PREF). The complex metabolism module explicitly accounts for a pyruvate intermediate and glucose/oxygen utilization is based on actual energy requirements for the given tick. The medium metabolism module maintains utilization based on actual energy requirements, but does not use a pyruvate intermediate. The simple metabolism module assumes utilization based on constant ATP production rate. Default parameter values are given in Supplementary Table 4.

Several stoichiometric ratios are defined:

- *S*_*glyc*_ = ATP produced per glucose from glycolysis (2 ATP/glucose)
- *S*_*oxphos*_ = ATP produced per pyruvate from oxidative phosphorylation (15 ATP/pyruvate)
- *S*_*PG*_ = pyruvate per glucose in glycolysis (2 pyruvate/glucose)
- *S*_*OP*_ = oxygen per pyruvate in oxidative phosphorylation (3 oxygen/pyruvate)

#### 4.3.1. Determine Nutrient Availability

At each tick (representing one minute), for each cell agent, the external glucose *G*_*ext*_ and oxygen *O*_*ext*_ are calculated from the environmental glucose G and oxygen O concentrations:

$$\begin{array}{l} G_{ext}^{t}=G^{t}\cdot V\\ O_{ext}^{t}=O^{t}\cdot V\cdot \rho \end{array}$$

where V is the volume of the hexagonal location (HEX_VOLUME) and ρ is the solubility of oxygen in tissue (OXY_SOLU_TISSUE).

#### 4.3.2. Consume Energy

Energy consumed *E*_*cons*_ is given by:

$$\begin{array}{l} E_{cons}=v\left(E_{0}+E_{pr}\cdot x_{pr}+E_{mi}\cdot x_{mi}\right) \end{array}$$

where *E*_0_ is basal energy consumption (BASAL_ENERGY), *E*_*pr*_ and *E*_*mi*_ are additional energy consumed for proliferation (PROLIF_ENERGY) and migration (MIGRA_ENERGY), respectively, and *x*_*pr*_ and *x*_*mi*_ are cell state flags (*mi* = migratory, *pr* = proliferative) which can be on (1) or off (0).

Energy requirement *E*_*req*_ for the current timepoint includes *E*_*cons*_ and any additional energy E requirement remaining from the previous time step:

$$\begin{array}{l} E_{req}=E_{cons}+E^{t-1} \end{array}$$

#### 4.3.3. Uptake Glucose

Internal glucose *G*_*int*_ increases by glucose uptake:

$$\begin{array}{l} G_{int}^{t}=G_{int}^{t-1}+G_{uptake} \end{array}$$

where glucose uptake *G*_*uptake*_ varies by module complexity.

For random metabolism:

$$\begin{array}{l} G_{uptake}=G_{ext}^{t}\cdot X_{GU} \end{array}$$

where *X*_*GU*_ = random number drawn from a uniform distribution *U*([0.005, 0.015]).

For simple metabolism:

$$\begin{array}{l} G_{uptake}=k_{U}\cdot \left(\frac{G_{ext}^{t}}{V}-\frac{G_{int}^{t-1}}{v}\right) \end{array}$$

where *k*_*U*_ = constant glucose uptake rate (CONS_GLUC_UPTAKE).

For medium metabolism:

$$\begin{array}{l} G_{uptake}=k_{P}\cdot v\cdot \left(\frac{G_{ext}^{t}}{V}-\frac{G_{int}^{t-1}}{v}\right)\cdot \left(\frac{1}{S_{avg}}\right) \end{array}$$

where *k*_*P*_ = ATP production rate (ATP_PRODUCTION_RATE) and *S*_*avg*_ = average ATP produced per glucose calculated as μ·*S*_*glyc*_ + (1 − μ)·*S*_*oxphos*_·*S*_*PG*_.

For complex metabolism:

$$\begin{array}{l} G_{uptake}=k_{G}\cdot A\cdot \left(\frac{G_{ext}^{t}}{V}-\frac{G_{int}^{t-1}}{v}\right) \end{array}$$

where *k*_*G*_ = glucose uptake rate (GLUC_UPTAKE_RATE) and *A* = cell agent surface area based on the cell volume.

#### 4.3.4. Calculate Nutrient Requirements

The amount of glucose required *G*_*req*_, amount of pyruvate required *P*_*req*_ (complex only), and oxygen uptake *O*_*uptake*_, can be calculated depending on module complexity.

For random metabolism:

$$\begin{array}{l} \;G_{req}^{glyc}=X_{GR}\\ O_{uptake}=O_{ext}^{t}\cdot X_{OU} \end{array}$$

where *X*_*GR*_ is a random number drawn from a uniform distribution *U*([0.2, 0.4]) and *X*_*OU*_ is a random number drawn from a uniform distribution *U*([0.2, 0.5]).

For simple metabolism:

$$\begin{array}{l} \;G_{req}^{glyc}=\frac{\mu \cdot \alpha }{S_{glyc}}\\ G_{req}^{oxphos}=\frac{\left(1-\mu \right)\cdot \alpha }{S_{oxphos}\cdot S_{PG}}\\ O_{uptake}=\text{min}\left(O_{ext},G_{req}^{oxphos}\cdot S_{PG}\cdot S_{OP}\right) \end{array}$$

where α is the constant ATP production rate (CONS_ATP_PRODUCTION).

For medium metabolism:

$$\begin{array}{l} \;G_{req}^{glyc}=\frac{\mu \cdot E_{req}}{S_{glyc}}\\ G_{req}^{oxphos}=\frac{\left(1-\mu \right)\cdot E_{req}}{S_{oxphos}\cdot S_{PG}}\\ O_{uptake}=\text{min}\left(O_{ext},G_{req}^{oxphos}\cdot S_{PG}\cdot S_{OP}\right) \end{array}$$

For complex metabolism:

$$\begin{array}{l} \;G_{req}=\frac{\mu \cdot E_{req}}{S_{glyc}}\\ \;P_{req}=\frac{\left(1-\mu \right)\cdot E_{req}}{S_{oxphos}}\\ O_{uptake}=\text{min}\left(O_{ext},P_{req}\cdot S_{OP}\right) \end{array}$$

#### 4.3.5. Generate Energy

Energy is generated through oxidative phosphorylation and glycolysis based on internal glucose or pyruvate, depending on the module complexity.

For oxidative phosphorylation with random, simple, and medium metabolism, the amount of glucose needed in terms of oxygen *G*_*O*_ is calculated as *G*_*O*_ = *O*_*uptake*_/(*S*_*PG*_·*S*_*OP*_).

If *G*_*int*_ > *G*_*O*_:

$$\begin{array}{l} E_{gen}^{oxphos}=G_{O}\cdot S_{oxphos}\cdot S_{PG}\\ \;G_{int}=G_{int}-G_{O} \end{array}$$

If *G*_*int*_ ≤ *G*_*O*_:

$$\begin{array}{l} E_{gen}^{oxphos}=G_{int}\cdot S_{oxphos}\cdot S_{PG}\\ \;G_{int}=0\\ O_{uptake}=G_{int}\cdot S_{PG}\cdot S_{OP} \end{array}$$

For oxidative phosphorylation with complex metabolism, the amount of pyruvate needed in terms of oxygen *P*_*O*_ is calculated as *P*_*O*_ = *O*_*uptake*_/*S*_*OP*_.

If *P*_*int*_ > *P*_*O*_:

$$\begin{array}{l} E_{gen}^{oxphos}=P_{O}\cdot S_{oxphos}\\ \;P_{int}=P_{int}-P_{O} \end{array}$$

If *P*_*int*_ ≤ *P*_*O*_:

$$\begin{array}{l} E_{gen}^{oxphos}=P_{int}\cdot S_{oxphos}\\ \;P_{int}=0\\ O_{uptake}=P_{int}\cdot S_{OP} \end{array}$$

For glycolysis with random, simple, and medium metabolism:

If $G_{int}>G_{req}^{glyc}$:

$$\begin{array}{l} E_{gen}^{glyc}=G_{req}^{glyc}\cdot S_{glyc}\\ \;G_{int}=G_{int}-G_{req}^{glyc} \end{array}$$

If $G_{int}\leq G_{req}^{glyc}$:

$$\begin{array}{l} E_{gen}^{glyc}=G_{int}\cdot S_{glyc}\\ \;G_{int}=0 \end{array}$$

For glycolysis with complex metabolism:

If *G*_*int*_ > *G*_*req*_:

$$\begin{array}{l} E_{gen}^{glyc}=G_{req}\cdot S_{glyc}\\ \;G_{int}=G_{int}-G_{req}\\ \;P_{int}=P_{int}+G_{req}\cdot S_{PG} \end{array}$$

If *G*_*int*_ ≤ *G*_*req*_:

$$\begin{array}{l} E_{gen}^{glyc}=G_{int}\cdot S_{glyc}\\ \;G_{int}=0\\ \;P_{int}=P_{int}+G_{int}\cdot S_{PG} \end{array}$$

Note that for complex and medium metabolism, between oxidative phosphorylation and glycolysis, additional glucose can be diverted through glycolysis to compensate for an energy deficit (E<0 and *G*_*int*_ > 0) in cases where there is not enough oxygen for complete oxidative phosphorylation. The two pathways do not occur sequentially in real systems so this step ensures that the glycolysis pathway can be used to produce energy under hypoxic conditions.

$$\begin{array}{l} G_{req}=\text{max}\left(G_{req},\frac{-\left(E-E_{cons}+E_{gen}^{oxphos}\right)}{S_{glyc}}\right) \end{array}$$

#### 4.3.6. Update Energy

The final energy level, for all complexities, is given by:

$$\begin{array}{l} E^{t}=E^{t-1}+E_{gen}-E_{cons} \end{array}$$

where $E_{gen}=E_{gen}^{oxphos}+E_{gen}^{glyc}$ for complex metabolism.

#### 4.3.7. Generate Cell Mass

Cells will generate cell mass *m* during (i) proliferation and (ii) size maintenance, depending on module complexity, when not under an energy deficit (E≥0). Cells use a fraction of their internal glucose and pyruvate *f*_*m*_ (FRAC_MASS) to produce cell mass. The cell aims to main a critical mass *m*_*crit*_.

For random metabolism where (*x*_*pr*_ = 1 and *m* < 2*m*_*crit*_):

$$\begin{array}{l} \;\Delta m=X_{U}\left[\frac{G_{int}}{\phi }\right]\\ \;G_{int}=G_{int}-X_{U}\cdot G_{int} \end{array}$$

where *X*_*U*_ is a random number drawn from a uniform distribution *U*([0, 1]) and ϕ is the glucose recovered from cell mass (MASS_TO_GLUC).

For simple metabolism where (*x*_*pr*_ = 1 and *m* < 2*m*_*crit*_ and *G*_*int*_ > *k*_*M*_·ρ·ϕ):

$$\begin{array}{l} \;\Delta m=k_{M}\cdot \rho \\ \;G_{int}=G_{int}-k_{M}\cdot \rho \cdot \phi \end{array}$$

where *k*_*M*_ is a constant growth rate (CONS_GROWTH_RATE) and ρ is cell density (CELL_DENSITY).

For medium metabolism where (*x*_*pr*_ = 1 and *m* < 2*m*_*crit*_) or (*m* < 0.99*m*_*crit*_):

$$\begin{array}{l} \;\Delta m=f_{m}\left[\frac{G_{int}}{\phi }\right]\\ \;G_{int}=G_{int}\cdot \left(1-f_{m}\right) \end{array}$$

For complex metabolism where (*x*_*pr*_ = 1 and *m* < 2*m*_*crit*_) or (*m* < 0.99*m*_*crit*_):

$$\begin{array}{l} \;\Delta m=f_{m}\left[\frac{\lambda \cdot G_{int}}{\phi }+\frac{\left(1-\lambda \right)\cdot P_{int}}{S_{PG}\cdot \phi }\right]\\ \;G_{int}=G_{int}\cdot \left(1-f_{m}\cdot \lambda \right)\\ \;P_{int}=P_{int}\cdot \left(1-f_{m}\cdot \left(1-\lambda \right)\right) \end{array}$$

where λ is the relative contribution of glucose and pyruvate to cell mass (RATIO_GLUC_TO_PYRU).

#### 4.3.8. Consume Cell Mass

For complex and medium metabolism, a cell consumes cell mass through autophagy (Glick et al., 2010) when (i) it is under an energy deficit and is larger than the minimum viable mass (E<0 and *m* > *m*_*min*_) or (ii) it is not under and energy deficit, is above its desired size, and it not proliferating (E≥0 and *m* > 1.01*m*_*crit*_ and *x*_*pr*_ = 1):

$$\begin{array}{l} \;m=m-k_{A}\\ G_{int}=G_{int}+k_{A}\cdot \phi \end{array}$$

where *m*_*min*_ is the minimum mass the cell agent tolerates (MIN_MASS_FRAC) and *k*_*A*_ is the rate of autophagy (AUTOPHAGY_RATE). Simple and random metabolism do not have a mechanism to consume mass.

#### 4.3.9. Update Cell and Environment

Cell volume is updated from cell mass *m* using cell density ρ as *v* = *m*/ρ. For complex metabolism, interval pyruvate is removed through conversion to lactate at rate *k*_*L*_ (LACTATE_RATE):

$$\begin{array}{l} P_{int}=\left(1-k_{L}\right)\cdot P_{int} \end{array}$$

The external glucose and oxygen environments are updated based on final uptake by the cell:

$$\begin{array}{l} G^{t+1}=G^{t}\cdot \left(1-\frac{G_{uptake}}{G_{ext}^{t}}\right)\\ O^{t+1}=O^{t}\cdot \left(1-\frac{O_{uptake}}{O_{ext}^{t}}\right) \end{array}$$

### 4.4. Signaling Modules

The complex signaling module is based on a published EGFR gene-protein interaction network (Athale et al., 2005; Zhang et al., 2007). The medium and simple signaling modules are further simplifications of this network. For the random signaling module, cells become migratory with a certain probability (MIGRA_PROB). Default parameter values are given in Supplementary Table 5.

At each tick (representing 1 min), for each agent, the external TGFα concentration is determined from the lattice. The system of equations is iteratively solved using a forward Euler method with time steps of 1 s. The external TGFα concentration in the lattice is then set to the new value. Cell agent state is defined by the relative fold change Δ in active PLCγ:

$$\begin{array}{l} \Delta =\frac{\text{max}\left({\left[\text{PLC}\gamma \right]}^{t},{\left[\text{PLC}\gamma \right]}^{t-1}\right)}{\text{min}\left({\left[\text{PLC}\gamma \right]}^{t},{\left[\text{PLC}\gamma \right]}^{t-1}\right)} \end{array}$$

where *t* is the current tick. Cells in an undefined state with Δ greater than migratory threshold θ (MIGRA_THRESHOLD) become migratory; otherwise they become proliferative:

$$\begin{array}{l} \left\{\begin{array}{ccc} \Delta >\theta : & x_{mi}=1 & x_{pr}=0\\ \Delta \leq \theta : & x_{mi}=0 & x_{pr}=1 \end{array}\right\} \end{array}$$

where *x* is the cell state flag (*mi* = migratory, *pr* = proliferative).

#### 4.4.1. Regulatory Weighting

Regulatory interactions are simplified into weights *w* of the following form:

$$\begin{array}{l} w_{k}=1\pm \frac{X_{i}}{\text{WK}+X_{i}} \end{array}$$

where *i* indicates the regulatory species, ± indicates an increase (+) or decrease (−) in rate, and WK is the corresponding weighting parameter given in Supplementary Table 5.

#### 4.4.2. Uptake and Transport

For simple signaling, extracellular TGFα (*X*_1_) forms a complex with EGFR and is internalized into cytoplasmic TGFα-EGFR (*X*_2_). Membrane EGFR is not explicitly considered. Inactive PLCγ (*X*_3_) converts to active PLCγ (*X*_4_) and vice versa (Athale et al., 2005; Zhang et al., 2007).

$$\begin{array}{l} \frac{dX_{1}}{dt}=k_{6}-k_{1}X_{1}w_{G}w_{C}-k_{3}X_{1}\\ \frac{dX_{2}}{dt}=k_{1}X_{1}w_{G}w_{C}-k_{2}X_{2}\\ \frac{dX_{3}}{dt}=k_{5}X_{4}-k_{4}\left(1-X_{4}\right)w_{P}\\ \frac{dX_{4}}{dt}=k_{4}\left(1-X_{4}\right)w_{P}-k_{5}X_{4} \end{array}$$

For medium signaling, extracellular TGFα (*X*_1_) and membrane EGFR (*X*_2_) form a TGFα-EGFR complex (*X*_3_) that autophosphorylates into p-TGFα-EGFR (*X*_4_) (Athale et al., 2005; Zhang et al., 2007). Unlike complex signaling, the translation of TGFα and EGFR are no longer explicitly included; TGFα secretion and EGFR insertion occur at constant rates. The complex is internalized into cytoplasmic TGFα-EGFR (*X*_5_), which can then dissociate. Inactive PLCγ (*X*_6_) converts to active PLCγ (*X*_7_) and vice versa (Athale et al., 2005; Zhang et al., 2007).

$$\begin{array}{l} \frac{dX_{1}}{dt}=k_{-1}X_{3}-k_{1}X_{1}X_{2}-k_{7}X_{1}+k_{11}\\ \frac{dX_{2}}{dt}=k_{-1}X_{3}-k_{1}X_{1}X_{2}-k_{6}X_{2}+k_{10}\\ \frac{dX_{3}}{dt}=2k_{1}X_{1}X_{2}-2k_{-1}X_{3}-k_{2}X_{3}w_{G}+k_{-2}X_{4}w_{C}-k_{3}X_{3}\\ \frac{dX_{4}}{dt}=k_{2}X_{3}w_{G}-k_{-2}X_{4}w_{C}-k_{4}X_{4}\\ \frac{dX_{5}}{dt}=k_{3}X_{3}+k_{4}X_{4}-k_{5}X_{5}\\ \frac{dX_{6}}{dt}=k_{9}X_{7}-k_{8}\left(1-X_{7}\right)w_{P}\\ \frac{dX_{7}}{dt}=k_{8}\left(1-X_{7}\right)w_{P}-k_{9}X_{7} \end{array}$$

For complex signaling, extracellular TGFα (*X*_1_) and membrane EGFR (*X*_2_) form a TGFα-EGFR complex (*X*_3_) that autophosphorylates into p-TGFα-EGFR (*X*_4_) (Athale et al., 2005; Zhang et al., 2007). The complex is internalized into cytoplasmic TGFα-EGFR (*X*_5_), which can then dissociate into cytoplasmic EGFR (*X*_6_) and TGFα (*X*_7_). Both EGFR and TGFα are translated from EGFR RNA (*X*_8_) and TGFα RNA (*X*_9_), respectively (Athale et al., 2005; Zhang et al., 2007). Inactive PLCγ (*X*_10_) converts to active PLCγ (*X*_11_) and vice versa (Athale et al., 2005; Zhang et al., 2007). EGFR RNA and TGFα RNA are generated from a nucleotide pool (*X*_12_) (Athale et al., 2005; Zhang et al., 2007).

$$\begin{array}{l} \frac{dX_{1}}{dt}=k_{-1}X_{3}-k_{1}X_{1}X_{2}+k_{9}X_{7}-k_{11}X_{1}\\ \frac{dX_{2}}{dt}=k_{-1}X_{3}-k_{1}X_{1}X_{2}+k_{8}X_{6}-k_{-8}X_{2}-k_{10}X_{2}\\ \frac{dX_{3}}{dt}=2k_{1}X_{1}X_{2}-2k_{-1}X_{3}-k_{2}X_{3}w_{G}+k_{-2}X_{4}w_{C}-k_{3}X_{3}\\ \frac{dX_{4}}{dt}=k_{2}X_{3}w_{G}-k_{-2}X_{4}w_{C}-k_{4}X_{4}\\ \frac{dX_{5}}{dt}=k_{3}X_{3}+k_{4}X_{4}+2k_{-5}X_{6}X_{7}-2k_{5}X_{5}\\ \frac{dX_{6}}{dt}=k_{5}X_{5}-k_{-5}X_{6}X_{7}+k_{14}X_{8}-k_{6}X_{6}-k_{8}X_{6}+k_{-8}X_{2}\\ \frac{dX_{7}}{dt}=k_{5}X_{5}-k_{-5}X_{6}X_{7}+k_{15}X_{9}-k_{7}X_{7}-k_{9}X_{7}\\ \frac{dX_{8}}{dt}=k_{16}X_{12}w_{E}-k_{18}X_{8}\\ \frac{dX_{9}}{dt}=k_{17}X_{12}w_{T}-k_{19}X_{9}\\ \frac{dX_{10}}{dt}=k_{13}X_{11}-k_{12}\left(1-X_{11}\right)w_{P}\\ \frac{dX_{11}}{dt}=k_{12}\left(1-X_{11}\right)w_{P}-k_{13}X_{11}\\ \frac{dX_{12}}{dt}=-k_{16}X_{12}w_{E}-k_{17}X_{12}w_{T}+k_{18}X_{8}+k_{19}X_{9} \end{array}$$

#### 4.4.3. Initial Concentrations and Regulatory Species

For simple metabolism, initial concentrations (in nM) are *X*_6_ = 0.333, and *X*_7_ = 0.667. Extracellular TGFα (*X*_1_) is given by CONC_TGF. All other species are initially at 0. Regulatory species are internal glucose (determined from the metabolism module) for *G*, *X*_4_ for *C*, and *X*_2_ for *P*.

For medium metabolism, initial concentrations (in nM) are *X*_2_ = 25, *X*_6_ = 0.333, and *X*_7_ = 0.667. Extracellular TGFα (*X*_1_) is given by CONC_TGF. All other species are initially at 0. Regulatory species are internal glucose (determined from the metabolism module) for *G*, *X*_7_ for *C*, and *X*_4_ for *P*.

For complex metabolism, initial concentrations (in nM) are *X*_2_ = 25, *X*_6_ = *X*_7_ = 5, *X*_8_ = *X*_9_ = 2.5, *X*_10_ = 0.333, *X*_11_ = 0.667, and *X*_12_ = 5. Extracellular TGFα (*X*_1_) is given by CONC_TGF. All other species are initially at 0. Regulatory species are internal glucose (determined from the metabolism module) for *G*, *X*_11_ for *C*, and *X*_4_ for *E*, *T*, and *P*.

### 4.5. Simulation Data Analysis

#### 4.5.1. Doubling Time

Doubling time is given by (*t*_*b*_ − *t*_*a*_)·ln2/ln(*N*_*b*_/*N*_*a*_) where *t* is time and *N* is the number of cells. Doubling time is calculated for each seed at 7 days (*a* = 0 and *b* = 7) across *n* = 50 replicates. Doubling time is also calculated by ln2/*r* where *r* is obtained by fitting an exponential curve *N* = *N*_0_exp(*t*·*r*) to each seed for the first 7 days across *n* = 50 replicates.

#### 4.5.2. Colony Diameter

Colony diameter *D* is calculated as the average of the diameter across the three hexagonal axes using $D=C[\max\left(u_{max}-u_{min}+1,0\right)$ + max(*v*_*max*_ − *v*_*min*_ + 1, 0) + max(*w*_*max*_ − *w*_*min*_ + 1, 0)]/3 where subscripts *max* and *min* refer to the maximum and minimum, respectively, of the given hexagonal coordinate across all cell locations at a given timepoint, and C is a scaling factor of 30 μm · hex^−1^ (HEX_SIZE). Colony diameter is calculated at each timepoint for *n* = 50 replicates.

#### 4.5.3. Cell Diameter

Assuming a cylindrical cell whose volume *v* is calculated as *v* = π*r*^2^*h* where *r* is radius and *h* is height, cell diameter *d* is given by $d=2\sqrt{v/\pi h}$ using an average height of *h* = 4.35, which is calculated as half the max cell height (MAX_HEIGHT).

#### 4.5.4. Fraction Occupancy

At a given seed and timepoint, the fraction occupancy at a radius *r* from the center is given by *n*/*N*_*r*_ where *n* is the number of agents of the state or population of interest and *N*_*r*_ is the maximum possible number of locations at radius *r* in a hexagonal grid. Note that fraction occupancy can exceed 1 as there can be more than a single agent per location.

#### 4.5.5. Relative Fraction Change

For simulations in which there are multiple populations, the relative change in the fraction of a given population is calculated as (*x* − *x*_0_)/*s* where *x* is the fraction of the population, *x*_0_ is the initial fraction of the population, and *s* is a scaling factor equal to 1 − *x*_0_ or *x*_0_ if the change (*x* − *x*_0_) is positive or negative, respectively. For simulations using the tissue context, the healthy background population is not included in the calculation.

### 4.6. Emergent Behavior Metrics

#### 4.6.1. Growth Rate

Growth rate quantifies the temporal emergence of colony diameter over time, in units of μm · day^−1^. For each time index *i* in [2, 2.5, ..., 14] days, a least squares linear fit between timepoints [1, 1.5, ..., *t*_*i*_] and colony diameters [*D*_1_, *D*_1.5_, ..., *D*_*i*_] is performed (Python, function polyfit from package numpy with degree of 1). The growth rate is taken as the slope of this line.

#### 4.6.2. Symmetry

Symmetry quantifies the spatial emergence of colony shape at a given timepoint, ranging from 0 (not symmetric) to 1 (perfectly symmetric). In hexagonal coordinates, the colony is perfectly symmetric if for each location (u,v,w), the corresponding five locations (-w,-u,-v), (v,w,u), (-u,-v,-w), (w,u,v), and (-v,-w,-u) are all occupied. For a given seed and timepoint, for each unique occupied location *i*, the number of unoccupied corresponding locations *n*_*i*_ is determined. Duplicate locations are not counted. Symmetry is calculated as:

$$\begin{array}{l} 1-\frac{1}{N}\overset{N}{\underset{i}{\sum }}\frac{n_{i}}{5} \end{array}$$

where *N* is the number of unique occupied locations.

#### 4.6.3. Cycle Length

Cycle length quantifies the parametric emergence of cell cycle length, in units of hours. Each cell agent tracks the number of ticks (minutes) between when it switches to a proliferative state and when it successfully divides to create a daughter cell agent. For a given seed and timepoint, cycle lengths are first averaged per agent, then averaged across all agents.

### 4.7. Data Fitting

#### 4.7.1. Colony Size

For Figure 2G, an equation relating number of cells *n*, cell diameter *d*, and colony diameter *D* given by:

$$\begin{array}{l} n=a\left(D^{b}/d^{c}\right) \end{array}$$

with parameters *a*, *b*, and *c* (Meyskens et al., 1984), was fit to simulated data using non-linear least squares (Python, function curve_fit from package scipy.optimize).

#### 4.7.2. Parameter Statistics

The average value *x* of each cell parameter across all cells at each timepoint is calculated for *n* = 20 replicates. The mean (μ) and standard deviation (σ) of these averages are estimated using:

$$\begin{array}{l} \mu =\frac{1}{n}\overset{n}{\underset{i=1}{\sum }}x_{i}\;\sigma =\sqrt{n^{-1}\overset{n}{\underset{i=1}{\sum }}{\left(x_{i}-\mu \right)}^{2}} \end{array}$$

The distribution of average parameter values across replicates at a given timepoint is compared to the initial distribution of averages at *t* = 0 using a t-test with paired samples (Python, function ttest_rel from package scipy.stats). The variance of average parameter values across replicates at a given timepoint is compared to the variance of the initial distribution at *t* = 0 using Levene's test (Python, function levene from package scipy.stats).

### 4.8. Case Study Simulations

First, we select three parameters to reflect common cancerous cell phenotypes: (i) crowding tolerance (MAX_HEIGHT), which captures a cell's tolerance for crowding (Supplementary Table 1); (ii) metabolic preference (META_PREF), which controls a cell's preference for using glycolysis over oxidative phosphorylation to produce energy (Supplementary Table 4); and (iii) migratory threshold (MIGRA_THRESHOLD), which governs a cell's tendency to migrate instead of proliferate (Supplementary Table 5). The crowding tolerance parameter quantifies the sensitivity of cells to contact inhibition, a phenomenon where cells stop growing even with sufficient nutrients when they reach a certain level of confluency (Swat et al., 2009). Cancerous cells exhibit reduced, or lack of, contact inhibition (Hanahan and Weinberg, 2011). The Warburg effect, in which cancerous cells predominantly produce energy through glycolysis rather than oxidative phosphorylation even in the presence of sufficient oxygen, is captured by the metabolic preference parameter (Heiden et al., 2009; Hanahan and Weinberg, 2011) Finally, cancer cell motility is an important factor in metastasis. We use the migratory threshold parameter to control the cell agent decision between migratory and proliferative states (Zhang et al., 2007).

Input options used to run the simulations are summarized in Supplementary Table 3.

#### 4.8.1. Case Study 1: Context

We perform a sensitivity analysis on these parameters by varying the parameter value +/− 100% in increments of 10%. For each parameter and modification, cells were seeded in isolation and simulated for 14 days with 20 replicates and timepoints taken every 12 h. Four representative populations were selected: A (crowding tolerance at +50% of baseline), B (metabolic preference at +50% of baseline), C (migratory threshold at −50% of baseline), X (all parameters at baseline). All four representative populations have the ability to exit quiescence without external stimulation; in contrast, the generic background population used for tissue context simulations is unable to exit quiescence without stimulation. These populations, and combinations thereof, were simulated in isolation (colony, representing an *in vitro* context) and in an environment containing a generic background cell population (tissue, representing an *in vivo* context). Simulations were run for 15 days with the representative populations introduced at *t* = 1 day. All simulations were run with 20 replicates with timepoints taken every 12 h.

#### 4.8.2. Case Study 2: Competition

A modified population is created by varying one of the three parameters (crowding tolerance, metabolic preference, and migratory threshold) between −50% and +50% in increments of 10%. This modified population is initialized into the simulation along with an unmodified, basal population in different ratios and simulated for 14 days. All simulations contain 20 replicates with timepoints taken every 12 h.

Note that we specifically focus on interactions between two populations, as is common with most co-culture studies (Goers et al., 2014). Including additional populations in our simulation is straightforward (one can introduce an additional cell agent or modify the parameters of an existing cell agent). However, including additional populations in an experimental setting is more difficult and may not necessarily form a more accurate representation of system; for example, a co-culture model of the blood-brain barrier system performed better than the mono- and tri-culture models (Hatherell et al., 2011; Goers et al., 2014). This counter-intuitive observation further motivates the need for a computational model with which to interrogate population interactions. In this case, our framework can be used to guide experimental design by identifying the minimal number of populations necessary to model a system.

#### 4.8.3. Case Study 3: Heterogeneity

Within the model, certain cell parameters (such as initial cell volume and age) are derived from a distribution. However, the internal cell parameters are constant among cell agents within a given population. To add heterogeneity to these parameters, each cell agent was modified to draw its parameter values from truncated normal distributions with means equal to the defined parameter values and variances dictated by a new heterogeneity parameter (HETEROGENEITY, Supplementary Table 1). Daughter cells of the agent use the parent parameter values as the mean of the truncated normal distributions from which it draws its parameter values, enabling clonal evolution in which population means can tend toward more “fit” values.

We vary the heterogeneity within representative cell populations and simulate their evolution in both colony and tissue contexts. For the colony context, the generic background population also contains heterogeneity *H*_0_, termed background heterogeneity, whose value is not necessarily equal to that of the representative populations. Simulations were run for 15 days with the representative populations introduced at *t* = 1 day. All simulations contain 20 replicates with timepoints taken every 12 h.

## Data Availability Statement

The datasets generated for this study are available on request to the corresponding author.

## Author Contributions

JY and NB: Conceptualization, Writing – Original Draft, and Writing – Review & Editing. JY: Software, Formal Analysis, and Visualization. NB: Supervision.

## Conflict of Interest

The authors declare that the research was conducted in the absence of any commercial or financial relationships that could be construed as a potential conflict of interest.
