# Supplementary material for: Agent-Based Models Predict Emergent Behavior of Heterogeneous Cell Populations in Dynamic Microenvironments
Source: Front Bioeng Biotechnol. 2020 Jun 11;8:249. doi: 10.3389/fbioe.2020.00249 (PMC7301008; doi:10.3389/fbioe.2020.00249)
Supplement: Supplementary file 1 [file Data_Sheet_1.pdf]

Supplementary Material

SUPPLEMENTARY FIGURES

Supplementary Figure 1. Agent-based model framework input and output 2
Supplementary Figure 2. Tissue cell implementation 3
Supplementary Figure 3. Metabolism and signaling module complexity 4
Supplementary Figure 4. Case study 1: Context 5
Supplementary Figure 5. Case study 2: Competition 6
Supplementary Figure 6. Case study 3: Heterogeneity 7

SUPPLEMENTARY TABLES

Supplementary Table 1. Sources and derivation for agent parameters 8
Supplementary Table 2. Sources and derivation for environment parameters 9
Supplementary Table 3. Input options used to run simulations 10
Supplementary Table 4. Sources and derivation for metabolism module parameters 14
Supplementary Table 5. Sources and derivation for signaling module parameters 15

SUPPLEMENTARY REFERENCES 16

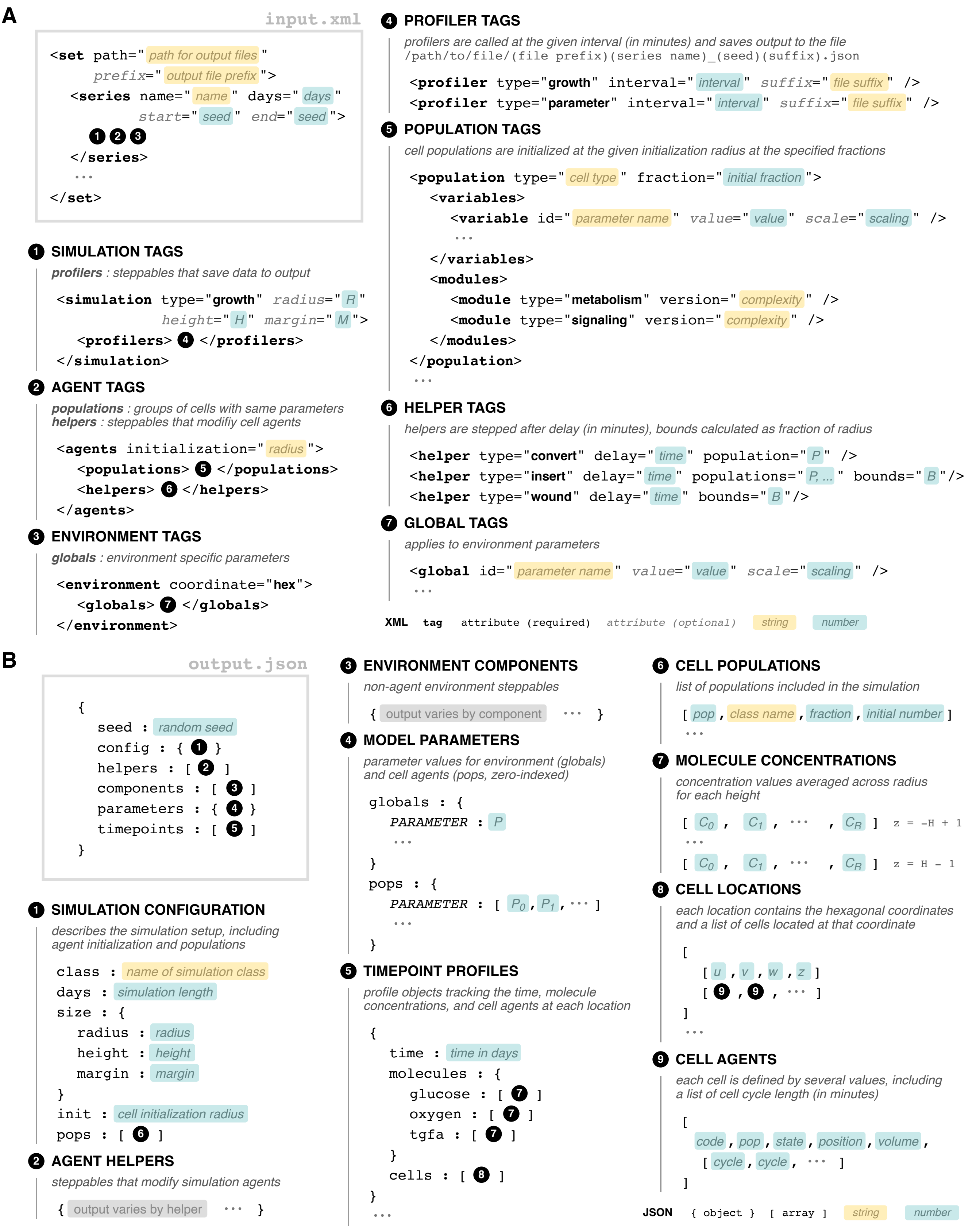

Supplementary Figure 1. Agent-based model framework input and output. (**A**) Diagram of the input XML structure. (**B**) Diagram of the output JSON structure for the `growth` profiler.

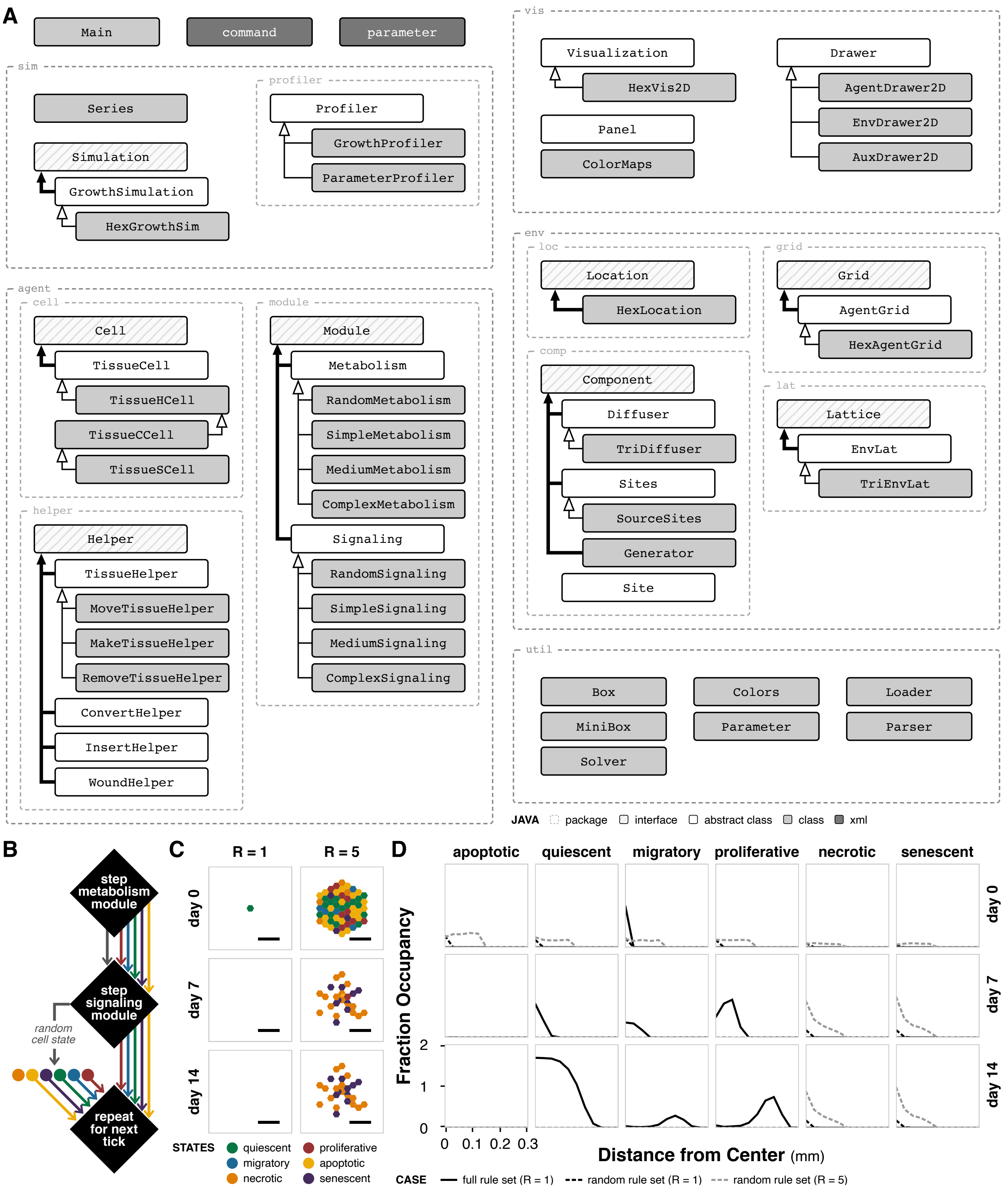

Supplementary Figure 2. Tissue cell implementation. (**A**) Diagram of the Java package structure for the tissue cell implementation of the framework. (**B**) Flowchart outlining agent rules for the random null model. (**C**) Spatial distribution of cell states for the random null model with different initializations for a single example replicate (random seed 0) at different timepoints. Scale bars represent 100 μm. (**D**) Average distribution of cell states as a function of distance from the center of the colony mass at selected timepoints across $n = 50$ replicates. Solid line and dotted lines indicate the full and random null rule sets, respectively.

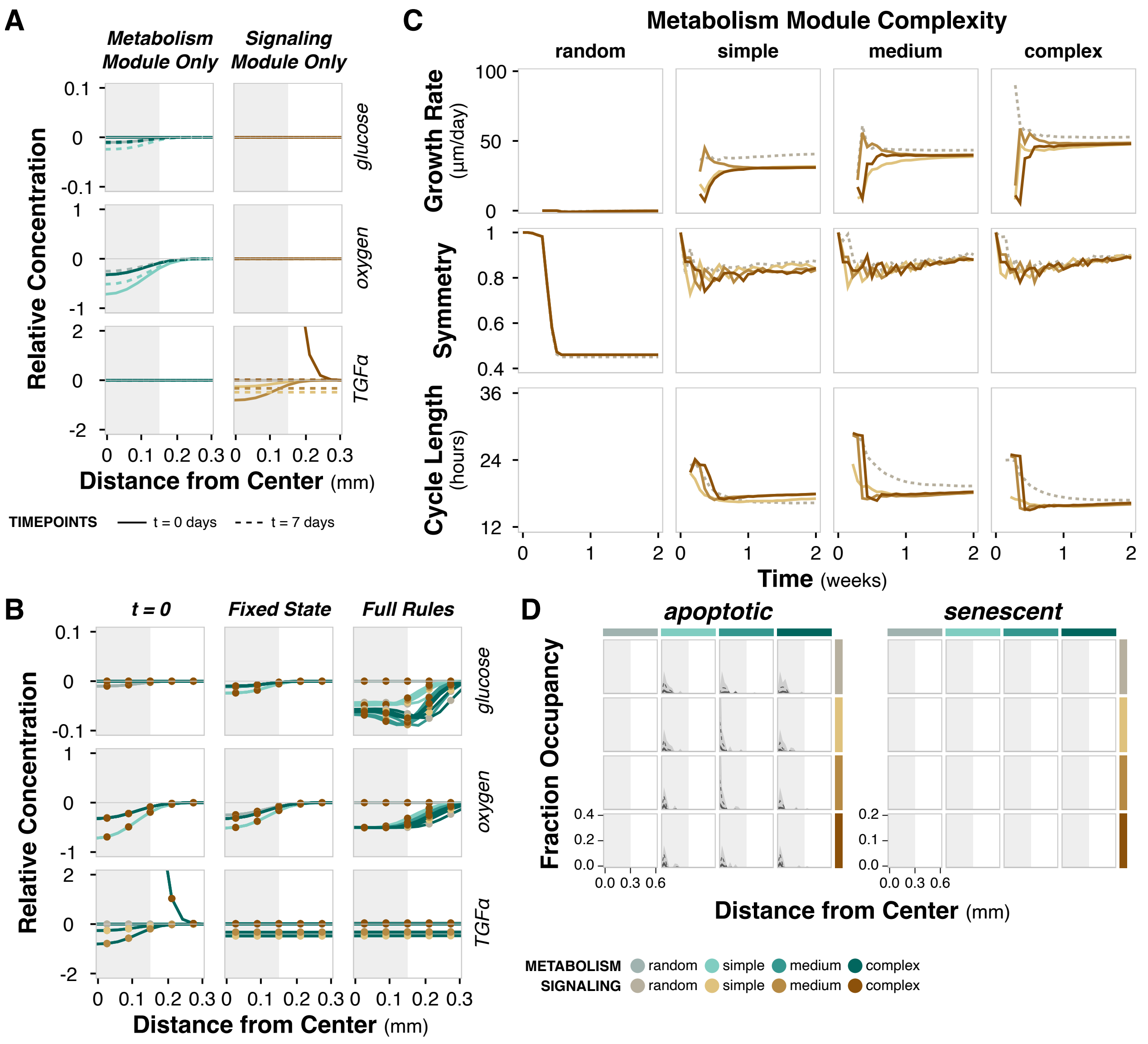

Supplementary Figure 3. Metabolism and signaling module complexity. (**A**) Average concentration of glucose, oxygen, and TGFα relative to source concentrations (Supplementary Table 2) for fixed state cell agents containing only a metabolism (*left*) or signaling (*right*) module for selected timepoints across $n = 20$ simulations. Agents initialized at a radius of 5 hexagons (gray shaded area). (**B**) Average concentration of glucose, oxygen, and TGFα relative to source concentrations (Supplementary Table 2) for fixed state cell agents (*middle*) or full rule set agents (*right*) containing both metabolism and signaling modules at timepoint $t = 7$ days across $n = 20$ simulations. Concentration at timepoint $t = 0$ (*left*) are the same between the two cases. Agents initialized at a radius of 5 hexagons (gray shaded area). (**C**) Time course of growth rate, symmetry, and cycle length for different complexities of the metabolism and signaling modules, grouped by metabolism module complexity. (**D**) Distribution of cell states as a function of distance from the center of the colony at $t = 2$ weeks. Solid line, dotted line, and shaded area denote the mean, standard deviation, and range across $n = 20$ replicates. Light gray rectangle is a visual reference for a distance of 0.3 mm from the center across all cell states.

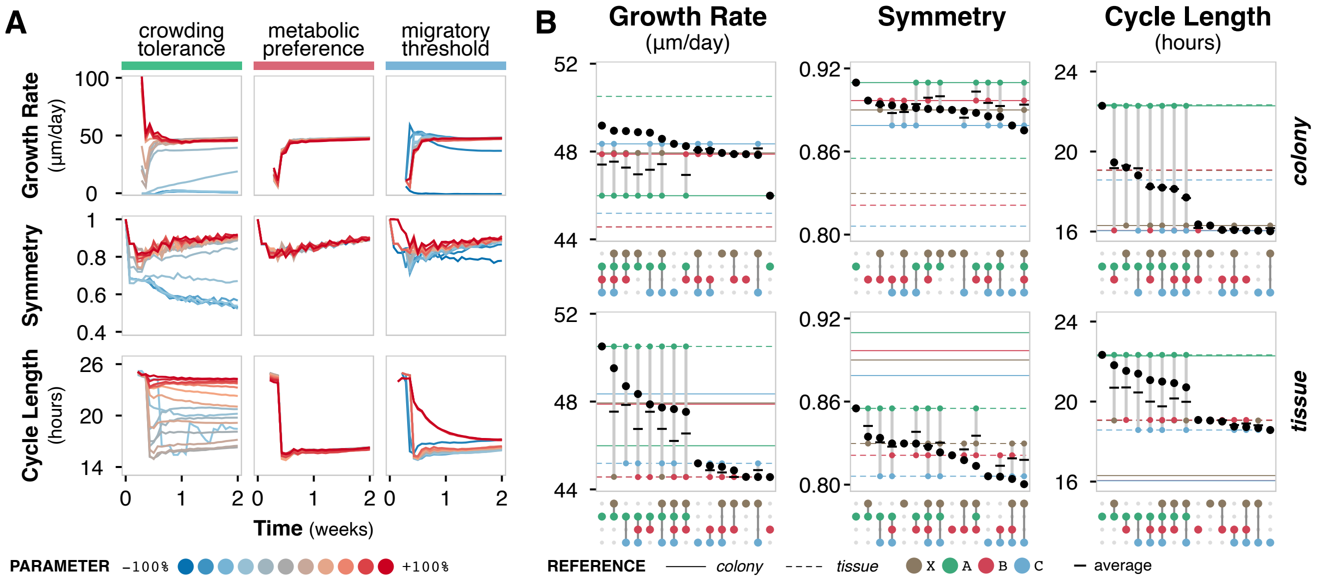

Supplementary Figure 4. Case study 1: Context. (**A**) Average metric values over time for different changes in parameters at the cell, metabolism, and signaling scales (crowding tolerance, metabolic preference, and migratory threshold, respectively). (**B**) Values of metrics for all combinations of the four representative populations over time under colony and tissue contexts. For each combination, the value of the metric for the constituent populations are given by smaller colored dots and the average is indicated by the black bar. Visual reference lines for the constituent populations are given by solid (colony) and dotted (tissue) lines.

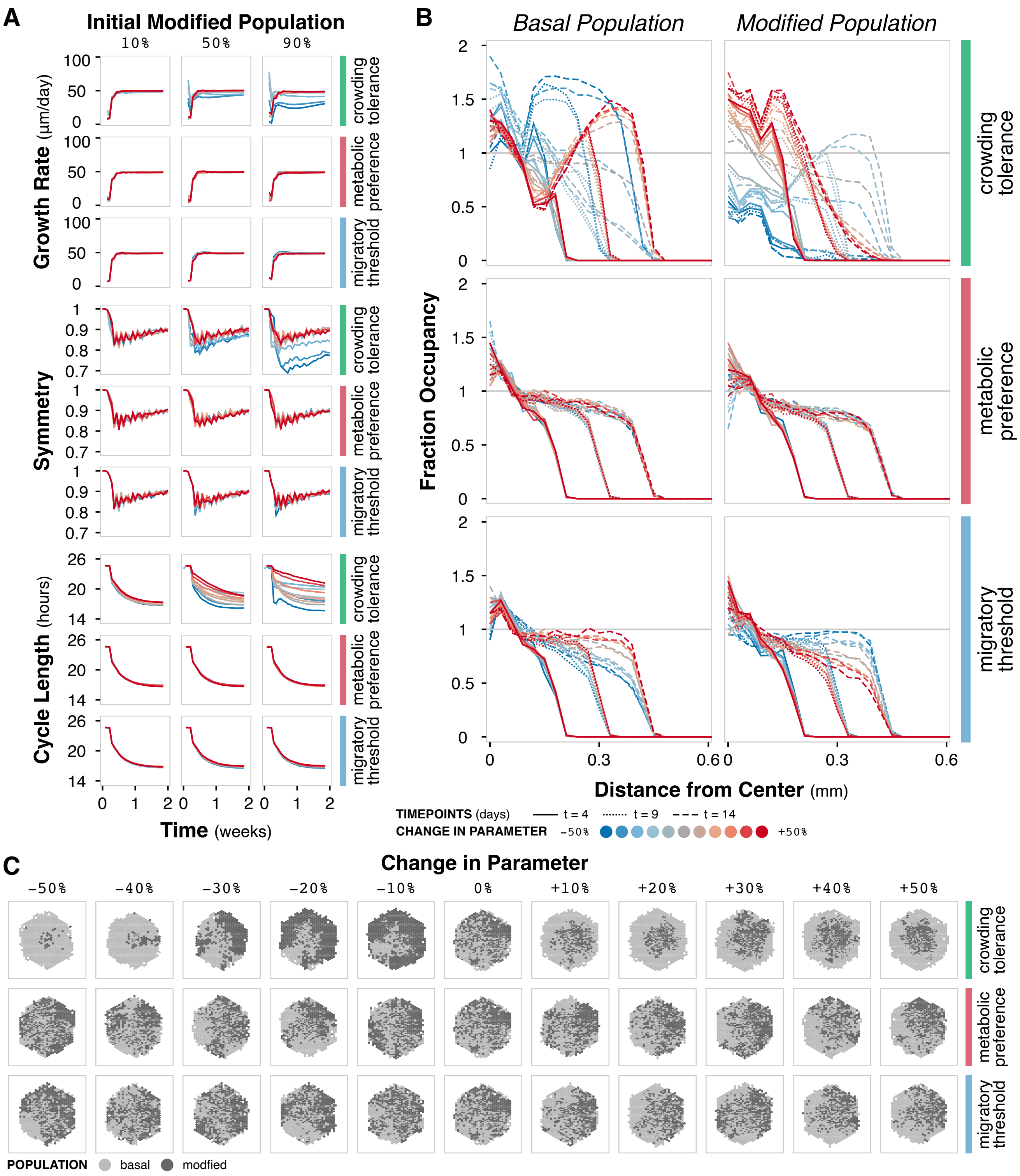

Supplementary Figure 5. Case study 2: Competition. (**A**) Average metric values over time for different changes in parameter and initial modified population. (**B**) Average fraction occupancy at different timepoints across different changes in parameter for simulations initialized with an equal mixture of the modified and basal cell populations. (**C**) Spatial distribution of populations at $t = 2$ weeks for random seed 0 across different changes in parameter for simulations initialized with an equal mixture of the modified and basal cell populations.

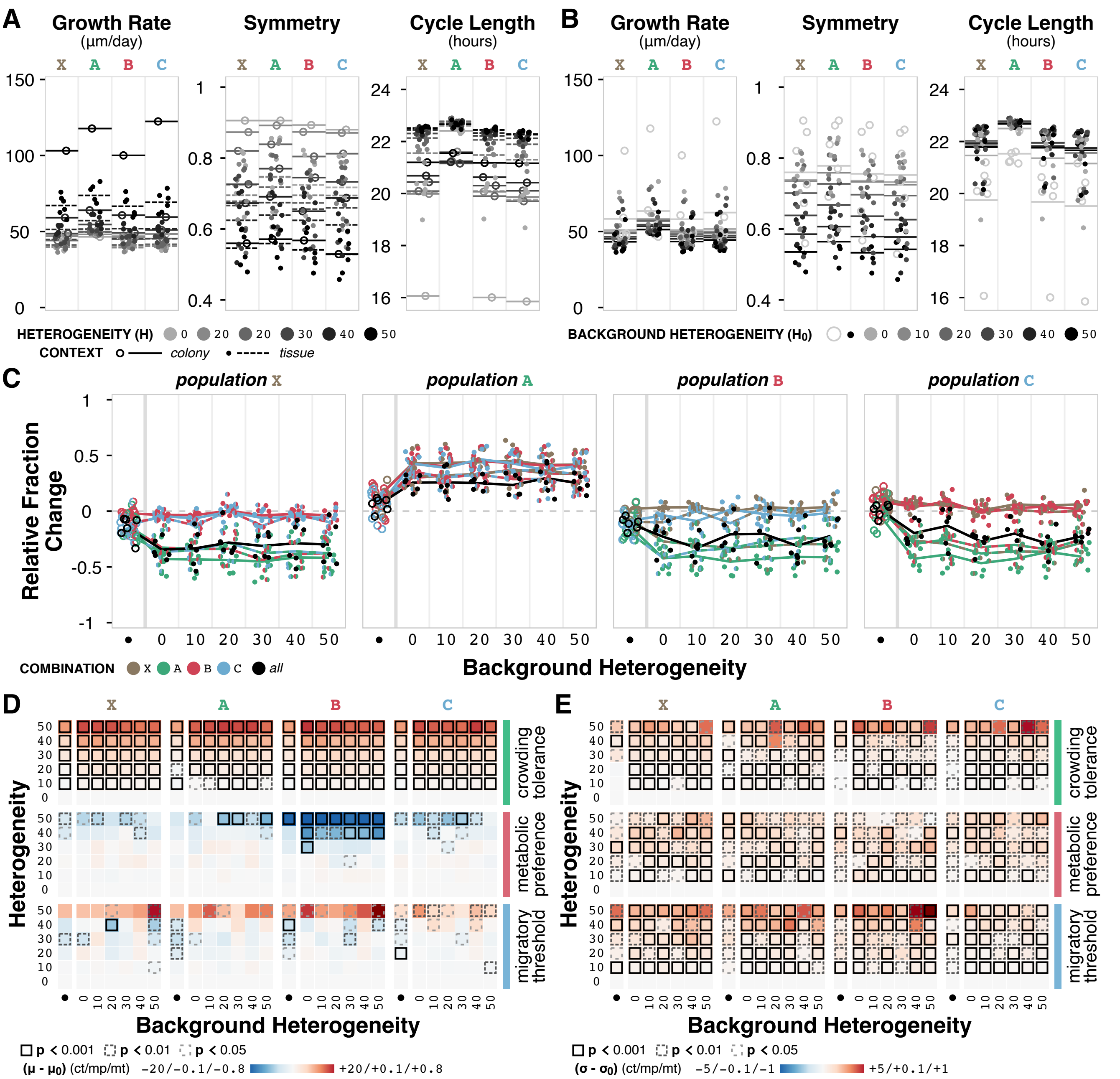

Supplementary Figure 6. Case study 3: Heterogeneity. (**A**) Metric values for the representative cell populations at $t = 2$ weeks colored by heterogeneity. Lines show the average value. (**B**) Metric values for the representative cell populations at $t = 2$ weeks colored by background heterogeneity. Lines show the average value. The colony context (no background population) is indicated by the empty circles. (**C**) Relative change in population fraction for each of the four representative populations for all combinations as a function of background heterogeneity at $t = 2$ weeks. Color indicates the other populations included in the simulation; black indicates all three other populations where included. Lines connect averages across values for different heterogeneities. The colony context is indicated by the bullet (•). (**D**) Heat maps of the change in mean (μ) of the average parameter value across $n = 20$ replicates at $t = 2$ weeks from the mean of the initial parameter distribution (μ_0). The colony context is indicated by the bullet (•). Borders indicate statistical significance for identical means between the two distributions. (**E**) Heat maps of the change in standard deviation (σ) of the average parameter value across $n = 20$ replicates at $t = 2$ weeks from the standard deviation of the initial parameter distribution (σ_0). The colony context is indicated by the bullet (•). Borders indicate statistical significance for equal variances between the two distribtions.

Supplementary Table 1. Sources and derivation for agent parameters.

Parameter	Code	Value	Source / Derivation
Cell life span average	DEATH_AGE_AVG ⋆	120960 min	1 – 120 days (Flindt, 2006)<br>10.7 ± 3.6, 15.1 years (Spalding et al., 2005)<br>Lifespan of cell greatly depends on type of cell. Use approximate average lifespan of 12 weeks.
Cell life span range	DEATH_AGE_RANGE	10080 min	*estimated*<br>Probability of apoptosis at lifespan given by cumulative normal distribution with $\mu =$ DEATH_AGE_AVG and $\sigma =$ DEATH_AGE_RANGE.
Apoptosis duration	DEATH_TIME	1080 min	12 – 24 hours (Saraste, 1999)<br>Use average of 18 hours.
Migration rate	MIGRA_RATE	0.24 $\mu\text{m} \cdot \text{min}^{-1}$	CHO cells: 10 – 20 $\mu\text{m} \cdot \text{hr}^{-1}$ (Palecek et al., 1997)<br>H69 cells: 0.26 $\mu\text{m} \cdot \text{min}^{-1}$ (Maulik et al., 2002)<br>hMEC cells: 8 – 16 $\mu\text{m} \cdot \text{hr}^{-1}$ (Smith et al., 2006)<br>Use average across all three sources.
Synthesis duration	SYNTHESIS_TIME	637 min	Human epithelial: 14, 10, 12 hours (Baserga, 1985)<br>Eukaryotic: 8.2, 9.0 hours (Weber et al., 2014)<br>HeLa S3: 10.5 hours (Sasaki et al., 1987)<br>Use average across all values for S phase.
Cell volume average	CELL_VOL_AVG	2250 μm^3	COS-7: 2016 ± 208.6 μm^3 (Bohil et al., 2006)<br>COS-7: 2.4 pL (Fujioka et al., 2006)<br>Approximate average.
Cell volume range	CELL_VOL_RANGE	200 μm^3	*estimated* (Bohil et al., 2006)
Max cell height (*crowding tolerance*)	MAX_HEIGHT ⋆	8.7 μm	$V = 2349, 3857$ μm^3 (Park et al., 2008)<br>$V = 2460, 3282, 2133$ μm^3 (Weiss et al., 2007)<br>$d = 21.2$ μm (Krombach et al., 1997)<br>$d = 19.1, 20.8, 16.0, 18.1, 16.1$ μm (Adams et al., 2014)<br>$d = 11.5, 12.4$ μm (Rosenbluth et al., 2006)<br>Convert volumes to diameter using $d = 2\sqrt{V/\pi h}$. Solve for cell height h such that h calculated from average cell volume (CELL_VOL_AVG) matches average diameter $\bar{d}$ (across sources) using $h = V/\pi(\bar{d}/2)^2$.
Cell density	CELL_DENSITY	0.00133 $\text{ng} \cdot \mu\text{m}^{-3}$	$2.48, 4.09$ ng (Park et al., 2008)<br>3 ng (Flamholz et al., 2014)<br>2.8 ng dry weight (Freitas, 1999)<br>For (Freitas, 1999), convert molecules to moles using Avogadro's number, then convert to mass using molecular weight. Use 3 ng and average cell volume (CELL_VOL_AVG) to calculate density.
Division potential	DIVISION_POTENTIAL ⋆	50 divisions	Based on Hayflick limit of 50 passages (Hayflick and Moorhead, 1961)
Energy threshold	ENERGY_THRESHOLD ⋆	-1000 fmol ATP	*estimated*<br>Cells unable to meet energy requirements accumulate energy deficit.
Necrotic fraction	NECRO_FRAC ⋆	0.5	*estimated*<br>Cells below ENERGY_THRESHOLD have a NECRO_FRAC% chance to become necrotic. Otherwise, they become apoptotic.
Sensecent fraction	SENES_FRAC ⋆	0.5	*estimated*<br>Cells above DIVISION_POTENTIAL have a SENES_FRAC% chance to become senescent. Otherwise, they become apoptotic.
Search Accuracy	ACCURACY ⋆	0.8	*estimated*<br>ACCURACY based on *search precision* (Mansury and Deisboeck, 2003).
Affinity	AFFINITY ⋆	0.5	*estimated*<br>Cells with higher affinity are more likely to migrate or proliferate toward the center of colony, representing cell-cell adhesion interactions.
Heterogeneity	HETEROGENEITY	0.0	*estimated*<br>All internal cell parameters (indicated by ⋆) are drawn from a normal distribution where $\mu =$ parameter value and $\sigma = H \cdot \mu$. Parameters META_PREF (Supplementary Table 3) and MIGRA_THRESHOLD (Supplementary Table 4) are also affected.

Supplementary Table 2. Sources and derivation for environment parameters.

Parameter	Code	Value	Source / Derivation
Hex Size	HEX_SIZE[a]	30 μm	Liver density ρ_L: 1.05 ± 0.013 g · mL^{-1} (Overmoyer et al., 1987)<br>Cell density ρ_C: $65 - 185 \times 10^6$ cells · g^{-1} (Wilson et al., 2003)<br>Spheroid diameter d_S: $400 - 500$ μm (Ghosh et al., 2005)<br>Cell count n_S: $1 - 3 \times 10^4$ cells (Ghosh et al., 2005)<br>Effective volume occupied by liver cells $V_e = (\rho_L \rho_C)^{-1}$ ranges from 5080 to 14820 μm^3 · cell^{-1}. Effective volume in spheroids $V_e = (4\pi/3)((d_S/2)^3/n_S)$ ranges from 1117 to 6545 μm^3 · cell^{-1}. There can be up to 6 cell agents per location in the model, giving effective volumes of 1130 to 6781 μm^3 · cell^{-1}.[b]
Hex Volume	HEX_VOLUME[a]	6781 μm^3	Given hex size H (HEX_SIZE), the volume of each location is $V = (3/2\sqrt{3})H^2 h$ where h is the max height (MAX_HEIGHT).
Glucose Diffusivity	DIFF_GLUC	39.0 μm^2 · s^{-1}	2.3×10^{-7} – 5.5×10^{-7}cm^2 · s^{-1} (Casciari et al., 1988)<br>Average within range.
Oxygen Diffusivity	DIFF_OXY	2000.0 μm^2 · s^{-1}	1.75×10^{-5} cm^2 · s^{-1} (Grote et al., 1977)<br>3.08×10^{-5} cm^2 · s^{-1} (Hou et al., 2010)<br>Approximate average between two sources.
TGFα Diffusivity	DIFF_TGF	51.8 μm^2 · s^{-1}	5.18×10^{-7} cm^2 · s^{-1} (Thorne et al., 2004)
Blood Glucose Concentration	CONC_GLUC	0.005 fmol · μm^{-3}	FPG < 5.6 mmol · L^{-1} (Powers, 2012)<br>5.1 mmol · L^{-1} (Giugliano et al., 2008)<br>FPG < 100 mg · dL^{-1} (Nathan et al., 2007)<br>Approximate average.
Blood Oxygen Partial Pressure	CONC_OXY	100 mmHg	100 mmHg (Biro, 2013)<br>12 kPa (90 mmHg) (McLellan and Walsh, 2004)
Initial TGFα Concentration	CONC_TGF	150 pg · cm^{-3}	159.8 pg · mL^{-1} (Chien et al., 1997)<br>147 ± 18 pg · mL^{-1} (Moskal et al., 1995)<br>Approximate average between two sources.
Oxygen solubility in tissue	OXY_SOLU _TISSUE	1.31×10^{-6} fmol O_2 · μm^{-3} · mmHg^{-1}	3.88×10^{-5} mL O_2 · cm^{-3} · mmHg^{-1} (Vadapalli et al., 2002)<br>2.81×10^{-5} mL O_2 · cm^{-3} · mmHg^{-1} (Vadapalli et al., 2002)<br>Average and convert to moles using ideal gas law assuming 1 atm and 310 K.

[a]The size of the hexagonal grid locations is fixed. They cannot be changed using global tags.
[b]Note that only one healthy (H) cell agent can occupy a given location, matching the effective volume range calculated from the healthy liver, whereas there can be up to 6 cancerous (C) and/or stem cell (S) agents, matching the effective volume range calculated from the tumor spheroid.

Supplementary Table 3. Input options used to run simulations. For clarity, wrapping `<set>` tags, `<series>` name attributes, and `<simulation>` tags are not shown. For each set, simulations were run for every combination of bold options grouped by square brackets and separated by pipes.

(A) DEFAULT *simulations use default parameters. The random case is run with modified code.*

<table>
<tr><th>Set</th><th>Input</th></tr>
<tr><td>Default (colony)</td><td><series start="0" end="51" days="15">
<agents initialization="0">
<populations>
<population type="C" fraction="0.0" />
</populations>
<helpers>
<helper type="insert" delay="1440" populations="0" bounds="0.01"/>
</helpers>
</agents>
</series></td></tr>
<tr><td>Default (tissue)</td><td><series start="0" end="51" days="15">
<agents initialization="FULL">
<populations>
<population type="H" fraction="1.0" />
</populations>
</agents>
</series></td></tr>
<tr><td>Default (<i>random</i>)</td><td><series start="0" end="50" days="15">
<agents initialization="0">
<populations>
<population type="C" fraction="0.0" />
</populations>
<helpers>
<helper type="insert" delay="1440" populations="0" bounds="[0.01|0.12]" />
</helpers>
</agents>
</series></td></tr>
</table>

(B) MODULE COMPLEXITY *simulations vary the complexity of the metabolism and signaling modules. The fixed state cases are run with modified code.*

Set	Input
Module Complexity	(see code below)
Module Complexity (*fixed state, both modules*)	(same as above, but using fixed state agent model)
Module Complexity (*fixed state, metabolism only*)	(see code below)
Module Complexity (*fixed state, signaling only*)	(see code below)

Module Complexity

```
<series start="0" end="20" days="15">
   <agents initialization="0">
      <populations>
         <population type="C" fraction="0.0">
            <modules>
               <module type="metabolism" version="[C|M|S|R]"/>
               <module type="signaling" version="[C|M|S|R]" />
            </modules>
         </population>
      </populations>
      <helpers>
         <helper type="insert" delay="1440" populations="0" bounds="0.12"/>
      </helpers>
   </agents>
</series>
```

Module Complexity (*fixed state, both modules*)

(same as above, but using fixed state agent model)

Module Complexity (*fixed state, metabolism only*)

```
<series start="0" end="20" days="15">
   <agents initialization="0">
      <populations>
         <population type="C" fraction="0.0">
            <modules>
               <module type="metabolism" version="[C|M|S|R]" />
               <module type="signaling" version="C" />
            </modules>
         </population>
      </populations>
      <helpers>
         <helper type="insert" delay="1440" populations="0" bounds="0.12"/>
      </helpers>
   </agents>
</series>
```

Module Complexity (*fixed state, signaling only*)

```
<series start="0" end="20" days="15">
   <agents initialization="0">
      <populations>
         <population type="C" fraction="0.0">
            <modules>
               <module type="metabolism" version="C" />
               <module type="signaling" version="[C|M|S|R]" />
            </modules>
         </population>
      </populations>
      <helpers>
         <helper type="insert" delay="1440" populations="0" bounds="0.12"/>
      </helpers>
   </agents>
</series>
```

(C) PARAMETER SENSITIVITY *simulations vary one of three parameter values +/−100%.*

Set	Input
Parameter Sensivitity	<series start="0" end="20" days="15"><br><agents initialization="0"><br><populations><br><population type="C" fraction="0.0"><br><variables><br><variable id="[**max_height**|**meta_pref**|**migra_threshold**]"<br>scale="[**0.0**|**0.1**|**0.2**|**0.3**|**0.4**|**0.5**|**0.6**|**0.7**|**0.8**|**0.9**|**1.0**| **1.1**|**1.2**|**1.3**|**1.4**|**1.5**|**1.6**|**1.7**|**1.8**|**1.9**|**2.0**]" /><br></variables><br></population><br></populations><br><helpers><br><helper type="insert" delay="1440" populations="0" bounds="0.12"/><br></helpers><br></agents><br></series>

(D) GROWTH CONTEXT *simulations include all combinations of four representative populations grown in isolation (colony) or with a generic background population (tissue).*

Set	Input
Growth Context (*colony*)	<series start="0" end="20" days="15"><br><agents initialization="0"><br><populations><br><population type="C" fraction="0.0" /><br><population type="C" fraction="0.0"><br><variables><br><variable id="max_height" scale="1.5" /><br></variables><br></population><br><population type="C" fraction="0.0"><br><variables><br><variable id="meta_pref" scale="1.5" /><br></variables><br></population><br><population type="C" fraction="0.0"><br><variables><br><variable id="migra_threshold" scale="0.5" /><br></variables><br></population><br></populations><br><helpers><br><helper type="insert" delay="1440" populations="[**0**|**1**|**2**|**3**| **0,1**|**0,2**|**0,3**|**1,2**|**1,3**|**2,3**|**0,1,2**|**0,1,3**|**1,2,3**|**0,2,3**|**0,1,2,3**]" bounds="0.12"/><br></helpers><br></agents><br></series>
Growth Context (*tissue*)	<series start="0" end="20" days="15"><br><agents initialization="FULL"><br><populations><br>(first four populations same as for colony context)<br><population type="H" fraction="1.0" /><br></populations><br>(helper same as for colony context)<br></agents><br></series>

(E) CELL COMPETITION *simulations include a population with a modified parameter and a basal population.*

<table>
<tr><th>Set</th><th>Input</th></tr>
<tr><td>Cell Competition</td><td><series start="0" end="20" days="14">
<agents initialization="5">
<populations>
<population type="C" fraction="[0.0|0.1|0.2|0.3|0.4|0.5|0.6|0.7|0.8|0.9|1.0]" />
<population type="C" fraction="[1.0|0.9|0.8|0.7|0.6|0.5|0.4|0.3|0.2|0.1|0.0]">
<variables>
<variable id="[max_height|meta_pref|migra_threshold]"
scale="[0.5|0.6|0.7|0.8|0.9|1.0|1.1|1.2|1.3|1.4|1.5]" />
</variables>
</population>
</populations>
</agents>
</series></td></tr>
</table>

(F) POPULATION HETEROGENEITY *simulations analogous to* GROWTH CONTEXT *but with heterogeneity.*

<table>
<tr><th>Set</th><th>Input</th></tr>
<tr><td>Population Heterogeneity (<i>colony</i>)</td><td><series start="0" end="20" days="15">
<agents initialization="0">
<populations>
<population type="C" fraction="0.0">
<variables>
<variable id="heterogeneity" value="[0.0|0.1|0.2|0.3|0.4|0.5]" />
</variables>
</population>
<population type="C" fraction="0.0">
<variables>
<variable id="max_height" scale="1.5" />
<variable id="heterogeneity" value=" (<i>same as population 0</i>) " />
</variables>
</population>
<population type="C" fraction="0.0">
<variables>
<variable id="meta_pref" scale="1.5" />
<variable id="heterogeneity" value=" (<i>same as population 0</i>) " />
</variables>
</population>
<population type="C" fraction="0.0">
<variables>
<variable id="migra_threshold" scale="0.5" />
<variable id="heterogeneity" value=" (<i>same as population 0</i>) " />
</variables>
</population>
</populations>
<helpers>
<helper type="insert" delay="1440" populations="[0|1|2|3|
0,1|0,2|0,3|1,2|1,3|2,3|0,1,2|0,1,3|1,2,3|0,2,3|0,1,2,3]" bounds="0.12"/>
</helpers>
</agents>
</series></td></tr>
<tr><td>Population Heterogeneity (tissue)</td><td><series start="0" end="20" days="15">
<agents initialization="FULL">
<populations>
(first four populations same as for colony context)
<population type="H" fraction="1.0">
<variables>
<variable id="heterogeneity" value="[0.0|0.1|0.2|0.3|0.4|0.5]" />
</variables>
</population>
</populations>
(helper same as for colony context)
</agents>
</series></td></tr>
</table>

Supplementary Table 4. Sources and derivation for metabolism module parameters.

Parameter	Code[a]	Value	Source / Derivation
Basal Energy Consumption	BASAL_ENERGY	0.001 fmol ATP $\cdot$ μm^{-3} $\cdot$ min^{-1}	68.4 ± 7.2 μmol ATP $\cdot$ g^{-1} $\cdot$ hr^{-1} (Buck et al., 1993)<br>1.7×10^{-11} mol ATP $\cdot$ cell^{-1} $\cdot$ day^{-1} (Kilburn et al., 1969)<br>10^7 ATP $\cdot$ cell^{-1} $\cdot$ s^{-1} (Flamholz et al., 2014)<br>Convert per cell using average volume (CELL_VOL_AVG) or per mass using cell density (CELL_DENSITY). Average is 0.002 fmol ATP $\cdot$ μm^{-3} $\cdot$ min^{-1}. Assume half for basal.
Proliferation Energy	PROLIF_ENERGY	0.001 fmol ATP $\cdot$ μm^{-3} $\cdot$ min^{-1}	See BASAL_ENERGY. Assume other half is for proliferation.
Migration Energy	MIGRA_ENERGY	0.0002 fmol ATP $\cdot$ μm^{-3} $\cdot$ min^{-1}	*estimated*
Mass to Glucose	MASS_TO_GLUC	694 fmol glucose $\cdot$ ng biomass^{-1}	Dry cell mass is 10% glucose carbon (Hosios et al., 2016)<br>Convert carbon mass to moles, and moles carbon to moles glucose (*i.e.* 6 moles C/mole glucose) to get 1388.9 fmol glucose $\cdot$ ng cell mass^{-1}. Multiply by fraction of glucose used for growth (FRAC_GROWTH_GLUC).
Metabolic preference	META_PREF (S·M·C)	0.3	70% ATP by oxidative phosphorylation (Zheng, 2012)<br>$20 \pm 21\%$ glycolytic ATP (Zu and Guppy, 2004)<br>$\approx 70\%$ ATP by oxidative phosphorylation (Fan et al., 2014)
Glucose Uptake Rate	GLUC_UPTAKE_RATE (C)	1.12 fmol glucose $\cdot$ μm^{-2} $\cdot$ min^{-1} $\cdot$ M^{-1}	0.75 nmol $\cdot$ μg protein^{-1} $\cdot$ hr^{-1}, 1 M (Lemons et al., 2010)<br>with ≈ 0.3 ng protein $\cdot$ cell^{-1} (Flamholz et al., 2014)<br>0.53 fmol $\cdot$ cell^{-1} $\cdot$ 3 min^{-1}, 0.1 mM (Noguchi et al., 1999)<br>200 ng $\cdot$ 10^6 cells^{-1} $\cdot$ min^{-1}, 3.2 mM (Noll et al., 2000)<br>Characteristic surface area $S = 4\pi\,(3V/4\pi)^{2/3}$ assuming spherical cell and average volume(CELL_VOL_AVG). Divide by surface area and glucose gradients, then average.
Production Rate of ATP	ATP_PRODUCTION_RATE (M)	8.927 fmol ATP $\cdot$ μm^{-3} $\cdot$ min^{-1} $\cdot$ M^{-1}	Use approximate ATP/glucose (ATP_PER_GLUCOSE). Convert glucose uptake rate (GLUC_UPTAKE_RATE) to per cell volume and moles glucose to moles ATP.
Constant Glucose Uptake	CONS_GLUC_UPTAKE (S)	929.9 fmol glucose $\cdot$ min^{-1} $\cdot$ M^{-1}	Convert glucose uptake rate (GLUC_UPTAKE_RATE) to constant by multiplying by characteristic cell surface area.
Constant Growth Rate	CONS_GROWTH_RATE (S)	2.819 μm^3 $\cdot$ min^{-1}	Man in vivo: $9 + 2$, $9 + 3$, $9 + 4$ hours (Baserga, 1985)<br>Eukaryotic: $8.9 + 5.6$ hours (Weber et al., 2014)<br>Human cell: $11 + 4 + 1$ hours (Cooper and Hausman, 2007)<br>Use average across all values for G1 + G2 + M phases and assume doubling of average volume (CELL_VOL_AVG).
Constant ATP Production	CONS_ATP_PRODUCTION (S)	4.9817 fmol ATP $\cdot$ cell^{-1} $\cdot$ min^{-1}	5×10^7 ATP $\cdot$ cell^{-1} $\cdot$ s^{-1} (Flamholz et al., 2014)
Minimum Mass Fraction	MIN_MASS_FRAC (M·C)	0.5	*estimated*<br>Mass to energy through autophagy until minimum fraction of starting mass.
Mass Production Fraction	FRAC_MASS (M·C)	0.25	*estimated*<br>Fraction of glucose (and pyruvate for C) converted to cell mass.
Ratio of Glucose to Pyruvate	RATIO_GLUC_TO_PYRU (C)	0.5	*estimated*<br>Preference for glucose over pyruvate for conversion to cell mass.
Autophagy Rate	AUTOPHAGY_RATE (M·C)	0.0001 ng $\cdot$ min^{-1}	Starvation: 0.82 autophagosomes $\cdot$ min^{-1} (Fass et al., 2006)<br>Autophagosomes: 300-900 nm diameter (Baba et al., 1997)<br>Assume spherical autophagosomes and average diameter of 600 nm and convert rate to mass basis using cell density (CELL_DENSITY).
Lactate Rate	LACTATE_RATE (C)	0.1 fmol lactate$\cdot$ fmol pyruvate^{-1}	37.2 μmol $\cdot$ 10^{10} cells^{-1} $\cdot$ hr^{-1} (Guppy et al., 1993)<br>Base case production of 0.675 fmol pyruvate $\cdot$ min^{-1} assuming quiescent cell of average volume (CELL_VOL_AVG) using basal energy (BASAL_ENERGY) with metabolic preference (META_PREF). Selected rate agrees with literature value of 0.062 fmol lactate $\cdot$ cell^{-1} $\cdot$ min^{-1}.

[a] Values in parentheses indicate only specific module complexities use the parameter (S = simple, M = medium, C = complex).

Supplementary Table 5. Sources and derivation for signaling module parameters.

**Parameter**[a]	**Code** (S/M/C)[b]			**Value**	**Source / Derivation**
Migratory threshold	`MIGRA_THRESHOLD`			3	*estimated* Fold change in PLCγ for migration.
Migratory probability[c]	`MIGRA_PROB`			0.05	*estimated*
TGFα-EGFR complex formation	·	`K1`	`K1`	3×10^{-3} nM$^{-1} \cdot$ s^{-1}	Zhang et al. (2007)
TGFα-EGFR complex dissociation	·	`K_1`	`K_1`	3.8×10^{-3} s^{-1}	Zhang et al. (2007)
TGFα-EGFR phosphorylation	·	`K2`	`K2`	1×10^{-3} s^{-1}	Zhang et al. (2007)
p-TGFα-EGFR dephosphorylation	·	`K_2`	`K_2`	1×10^{-6} s^{-1}	Zhang et al. (2007)
membrane TGFα-EGFR internalization	·	`K3`	`K3`	5×10^{-5} s^{-1}	Zhang et al. (2007)
membrane p-TGFα-EGFR internalization	·	`K4`	`K4`	5×10^{-5} s^{-1}	Zhang et al. (2007)
cytoplasmic TGFα-EGFR dissociation	·	`K5`	`K5`	1×10^{-2} s^{-1}	Zhang et al. (2007)
cytoplasmic TGFα-EGFR association	·	·	`K_5`	1.4×10^{-5} nM$^{-1} \cdot$ s^{-1}	Zhang et al. (2007)
cytoplasmic EGFR protein degradation	·	`K6`	`K6`	1.67×10^{-4} s^{-1}	Zhang et al. (2007)
cytoplasmic TGFα protein degradation	·	`K7`	`K7`	1.67×10^{-4} s^{-1}	Zhang et al. (2007)
cytoplasmic EGFR insertion	·	·	`K8`	5×10^{-3} s^{-1}	Zhang et al. (2007)
membrane EGFR internalization	·	·	`K_8`	5×10^{-5} s^{-1}	Zhang et al. (2007)
TGFα insertion and secretion	·	·	`K9`	1 s^{-1}	Zhang et al. (2007)
TGFα-EGFR complex formation and internalization	`K1`	·	·	3×10^{-3} s^{-1}	Zhang et al. (2007)
membrane EGFR degradation	`K2`	·	`K10`	1×10^{-4} s^{-1}	*estimate*
extracellular TGFα degradation	`K3`	·	`K11`	1×10^{-2} s^{-1}	*estimate*
PLCγ activation	`K4`	`K8`	`K12`	1×10^{-1} s^{-1}	Zhang et al. (2007)
PLCγ inactivation	`K5`	`K9`	`K13`	5×10^{-2} s^{-1}	Zhang et al. (2007)
EGFR RNA translation	·	·	`K14`	1.23×10^{-5} s^{-1}	5 molec $\cdot$ min^{-1} Athale et al. (2005)[d]
TGFα RNA translation	·	·	`K15`	1.23×10^{-5} s^{-1}	5 molec $\cdot$ min^{-1} Athale et al. (2005)[d]
EGFR RNA transcription	·	·	`K16`	5.34×10^{-6} s^{-1}	2.17 molec $\cdot$ min^{-1} Athale et al. (2005)[d]
TGFα RNA transcription	·	·	`K17`	2.95×10^{-5} s^{-1}	12 molec $\cdot$ min^{-1} Athale et al. (2005)[d]
EGFR RNA degradation	·	·	`K18`	2.95×10^{-9} s^{-1}	1.2×10^{-3} molec $\cdot$ min^{-1} Athale et al. (2005)[d]
TGFα RNA degradation	·	·	`K19`	2.95×10^{-9} s^{-1}	1.2×10^{-3} molec $\cdot$ min^{-1} Athale et al. (2005)[d]
EGFR RNA translation and insertion	·	`K10`	·	6.15×10^{-5} nM $\cdot$ s^{-1}	5 molec $\cdot$ min^{-1} Athale et al. (2005)[d]
TGFα RNA translation and secretion	`K6`	`K11`	·	6.15×10^{-5} nM $\cdot$ s^{-1}	5 molec $\cdot$ min^{-1} Athale et al. (2005)[d]
TGFα-EGFR phosphorylation by glucose	`WG`[e]	`WG`	`WG`	200	*estimated*
EGFR translation by p-TGFα-EGFR	·	·	`WE`	2	*estimated*
TGFα translation by p-TGFα-EGFR	·	·	`WT`	2	*estimated*
PLCγ activation by p-TGFα-EGFR	`WP`[e]	`WP`	`WP`	5	*estimated*
TGFα-EGFR dephosphorylation by active PLCγ	`WC`[e]	`WC`	`WC`	1	*estimated*

[a] Signaling parameters are defined within the specific signaling module class, rather than at the `parameter.xml` level. They cannot be changed using `variable` tags except for `MIGRA_THRESHOLD` and `MIGRA_PROB`.
[b] Columns indicate the parameter is only used by specific module complexities (S = simple, M = medium, C = complex).
[c] Random signaling module only.
[d] Convert molecules to concentration using 1355 molec $\cdot$ nM^{-1} calculated from Avogadro's constant and average cell volume (`CELL_VOL_AVG`). Assume basis of 5 nM nucleotide pool for complex signaling.
[e] Simple signaling does not account for phosphorylation. Instead, `WG` is TGFα-EGFR by glucose, `WP` is PLCγ activation by TGFα-EGFR, and `WC` is TGFα-EGFR by active PLCγ.

REFERENCES

Adams, D. L., Zhu, P., Makarova, O. V., Martin, S. S., Charpentier, M., Chumsri, S., et al. (2014). The systematic study of circulating tumor cell isolation using lithographic microfilters. *RSC Advances* 4, 4334–4342. doi:10.1039/c3ra46839a

Athale, C., Mansury, Y., and Deisboeck, T. S. (2005). Simulating the impact of a molecular 'decision-process' on cellular phenotype and multicellular patterns in brain tumors. *Journal of Theoretical Biology* 233, 469–481. doi:10.1016/j.jtbi.2004.10.019

Baba, M., Osumi, M., Scott, S. V., Klionsky, D. J., and Ohsumi, Y. (1997). Two distinct pathways for targeting proteins from the cytoplasm to the vacuole/lysosome. *The Journal of Cell Biology* 139, 1687–1695. doi:10.1083/jcb.139.7.1687

Baserga, R. (1985). *Table 1.2: Cell cycle times* (Cambridge, MA: Harvard University Press). 18

Biro, G. P. (2013). *From the Atmosphere to the Mitochondrion: The Oxygen Cascade* (Springer Berlin Heidelberg). 27–51

Bohil, A. B., Robertson, B. W., and Cheney, R. E. (2006). Myosin-x is a molecular motor that functions in filopodia formation. *Proceedings of the National Academy of Sciences* 103, 12411–12416. doi:10.1073/pnas.0602443103

Buck, L. T., Hochachka, P. W., Schon, A., and Gnaiger, E. (1993). Microcalorimetric measurement of reversible metabolic suppression induced by anoxia in isolated hepatocytes. *American Journal of Physiology - Regulatory, Integrative and Comparative Physiology* 265, R1014–R1019. doi:10.1152/ajpregu.1993.265.5.r1014

Casciari, J. J., Sotirchos, S. V., and Sutherland, R. M. (1988). Glucose diffusivity in multicellular tumor spheroids. *Cancer Research* 48, 3905–3909

Chien, C.-H., Huang, C.-C., Lin, Y.-H., Shen, J., and Chow, S.-N. (1997). Detection of serum transforming growth factor-α in patients of primary epithelial ovarian cancers by enzyme immunoassay. *Gynecologic Oncology* 66, 405–410. doi:10.1006/gyno.1997.4794

Cooper, G. M. and Hausman, R. E. (2007). *Chapter 16. The Cell Cycle* (Washington, D.C.: ASM Press). 650

Fan, J., Kamphorst, J. J., Mathew, R., Chung, M. K., White, E., Shlomi, T., et al. (2014). Glutamine-driven oxidative phosphorylation is a major ATP source in transformed mammalian cells in both normoxia and hypoxia. *Molecular Systems Biology* 9, 712–712. doi:10.1038/msb.2013.65

Fass, E., Shvets, E., Degani, I., Hirschberg, K., and Elazar, Z. (2006). Microtubules support production of starvation-induced autophagosomes but not their targeting and fusion with lysosomes. *Journal of Biological Chemistry* 281, 36303–36316. doi:10.1074/jbc.m607031200

Flamholz, A., Phillips, R., and Milo, R. (2014). The quantified cell. *Molecular Biology of the Cell* 25, 3497–3500. doi:10.1091/mbc.e14-09-1347

Flindt, R. (2006). *Table 4.1.1: Life span of cells in selected organs of the human body* (Berlin, Germany: Springer-Verlag Berlin Heidelberg). 204

Freitas, R. (1999). *Table 3.2. Estimated Gross Molecular Contents of a Typical 20-micron Human Cell* (Georgetown, TX: Landes Bioscience)

Fujioka, A., Terai, K., Itoh, R. E., Aoki, K., Nakamura, T., Kuroda, S., et al. (2006). Dynamics of the ras/ERK MAPK cascade as monitored by fluorescent probes. *Journal of Biological Chemistry* 281, 8917–8926. doi:10.1074/jbc.m509344200

Ghosh, S., Spagnoli, G. C., Martin, I., Ploegert, S., Demougin, P., Heberer, M., et al. (2005). Three-dimensional culture of melanoma cells profoundly affects gene expression profile: A high density oligonucleotide array study. *Journal of Cellular Physiology* 204, 522–531. doi:10.1002/jcp.20320

Giugliano, D., Ceriello, A., and Esposito, K. (2008). Glucose metabolism and hyperglycemia. *The American Journal of Clinical Nutrition* 87, 217S–222S. doi:10.1093/ajcn/87.1.217s

Grote, J., Süsskind, R., and Vaupel, P. (1977). Oxygen diffusivity in tumor tissue (ds-carcinosarcoma) under temperature conditions within the range of 20-40°c. *Pflügers Archive* 372, 37–42

Guppy, M., Greiner, E., and Brand, K. (1993). The role of the crabtree effect and an endogenous fuel in the energy metabolism of resting and proliferating thymocytes. *European Journal of Biochemistry* 212, 95–99. doi:10.1111/j.1432-1033.1993.tb17637.x

Hayflick, L. and Moorhead, P. (1961). The serial cultivation of human diploid cell strains. *Experimental Cell Research* 25, 585–621. doi:10.1016/0014-4827(61)90192-6

Hosios, A. M., Hecht, V. C., Danai, L. V., Johnson, M. O., Rathmell, J. C., Steinhauser, M. L., et al. (2016). Amino acids rather than glucose account for the majority of cell mass in proliferating mammalian cells. *Developmental Cell* 36, 540–549. doi:10.1016/j.devcel.2016.02.012

Hou, C., Gheorghiu, S., Huxley, V. H., and Pfeifer, P. (2010). Reverse engineering of oxygen transport in the lung: Adaptation to changing demands and resources through space-filling networks. *PLoS Computational Biology* 6, e1000902. doi:10.1371/journal.pcbi.1000902

Kilburn, D. G., Lilly, M. D., and Webb, F. C. (1969). The energetics of mammalian cell growth. *Journal of Cell Science* 4, 645–654

Krombach, F., Munzing, S., Allmeling, A.-M., Gerlach, J. T., Behr, J., and Dorger, M. (1997). Cell size of alveolar macrophages: An interspecies comparison. *Environmental Health Perspectives* 105, 1261. doi:10.2307/3433544

Lemons, J. M. S., Feng, X.-J., Bennett, B. D., Legesse-Miller, A., Johnson, E. L., Raitman, I., et al. (2010). Quiescent fibroblasts exhibit high metabolic activity. *PLoS Biology* 8, e1000514. doi:10.1371/journal.pbio.1000514

Mansury, Y. and Deisboeck, T. S. (2003). The impact of "search precision" in an agent-based tumor model. *Journal of Theoretical Biology* 224, 325–337. doi:10.1016/s0022-5193(03)00169-3

Maulik, G., Madhiwala, P., Brooks, S., Ma, P. C., Kijima, T., Tibaldi, E. V., et al. (2002). Activated c-met signals through PI3k with dramatic effects on cytoskeletal functions in small cell lung cancer. *Journal of Cellular and Molecular Medicine* 6, 539–553. doi:10.1111/j.1582-4934.2002.tb00453.x

McLellan, S. and Walsh, T. (2004). Oxygen delivery and haemoglobin. *Continuing Education in Anaesthesia Critical Care & Pain* 4, 123–126. doi:10.1093/bjaceaccp/mkh033

Moskal, T. L., Huang, S., Ellis, L. M., Fritsche, H. A., and Chakrabarty, S. (1995). Serum levels of transforming growth factor alpha in gastrointestinal cancer patients. *Cancer Epidemiology and Prevention Biomarkers* 4, 127–131

Nathan, D. M., Davidson, M. B., DeFronzo, R. A., Heine, R. J., Henry, R. R., Pratley, R., et al. (2007). Impaired fasting glucose and impaired glucose tolerance: Implications for care. *Diabetes Care* 30, 753–759. doi:10.2337/dc07-9920

Noguchi, Y., Sato, S., Marat, D., Doi, C., Yoshikawa, T., Saito, A., et al. (1999). Glucose uptake in the human gastric cancer cell line, MKN28, is increased by insulin stimulation. *Cancer Letters* 140, 69–74. doi:10.1016/s0304-3835(99)00054-3

Noll, T., Mühlensiepen, H., Engels, R., Hamacher, K., Papaspyrou, M., Langen, K.-J., et al. (2000). A cell-culture reactor for the on-line evaluation of radiopharmaceuticals: Evaluation of the lumped constant of fdg in human glioma cells. *Journal of Nuclear Medicine* 41, 556–564

Overmoyer, B. A., Mclaren, C. E., and Brittenham, G. M. (1987). Uniformity of liver density and nonheme (storage) iron distribution. *Archives of Pathology and Laboratory Medicine* 111, 549–554

Palecek, S. P., Loftus, J. C., Ginsberg, M. H., Lauffenburger, D. A., and Horwitz, A. F. (1997). Integrin-ligand binding properties govern cell migration speed through cell-substratum adhesiveness. *Nature* 385, 537–540. doi:10.1038/385537a0

Park, K., Jang, J., Irimia, D., Sturgis, J., Lee, J., Robinson, J. P., et al. (2008). 'living cantilever arrays' for characterization of mass of single live cells in fluids. *Lab on a Chip* 8, 1034. doi:10.1039/b803601b

Powers, A. C. (2012). *Chapter 344. Diabetes Mellitus* (New York, NY: The McGraw-Hill Companies). 2968–3002

Rosenbluth, M. J., Lam, W. A., and Fletcher, D. A. (2006). Force microscopy of nonadherent cells: A comparison of leukemia cell deformability. *Biophysical Journal* 90, 2994–3003. doi:10.1529/biophysj.105.067496

Saraste, A. (1999). Morphologic criteria and detection of apoptosis. *Herz* 24, 189–195

Sasaki, K., Murakami, T., and Takahashi, M. (1987). A rapid and simple estimation of cell cycle parameters by continuous labeling with bromodeoxyuridine. *Cytometry* 8, 526–528. doi:10.1002/cyto.990080514

Smith, J. T., Elkin, J. T., and Reichert, W. M. (2006). Directed cell migration on fibronectin gradients: Effect of gradient slope. *Experimental Cell Research* 312, 2424–2432. doi:10.1016/j.yexcr.2006.04.005

Spalding, K. L., Bhardwaj, R. D., Buchholz, B. A., Druid, H., and Frisén, J. (2005). Retrospective birth dating of cells in humans. *Cell* 122, 133–143. doi:10.1016/j.cell.2005.04.028

Thorne, R. G., Hrabětová, S., and Nicholson, C. (2004). Diffusion of epidermal growth factor in rat brain extracellular space measured by integrative optical imaging. *Journal of Neurophysiology* 92, 3471–3481. doi:10.1152/jn.00352.2004

Vadapalli, A., Goldman, D., and Popel, A. S. (2002). Calculations of oxygen transport by red blood cells and hemoglobin solutions in capillaries. *Artificial Cells, Blood Substitutes, and Biotechnology* 30, 157–188. doi:10.1081/bio-120004338

Weber, T. S., Jaehnert, I., Schichor, C., Or-Guil, M., and Carneiro, J. (2014). Quantifying the length and variance of the eukaryotic cell cycle phases by a stochastic model and dual nucleoside pulse labelling. *PLoS Computational Biology* 10, e1003616. doi:10.1371/journal.pcbi.1003616

Weiss, E., Wehner, F., and Lemor, R. (2007). *Measuring Cell Volume Regulation with Time Resolved Acoustic Microscopy* (Dordrecht, Netherlands: Springer Netherlands). 73–80

Wilson, Z. E., Rostami-Hodjegan, A., Burn, J. L., Tooley, A., Boyle, J., Ellis, S. W., et al. (2003). Inter-individual variability in levels of human microsomal protein and hepatocellularity per gram of liver. *British Journal of Clinical Pharmacology* 56, 433–440. doi:10.1046/j.1365-2125.2003.01881.x

Zhang, L., Athale, C. A., and Deisboeck, T. S. (2007). Development of a three-dimensional multiscale agent-based tumor model: Simulating gene-protein interaction profiles, cell phenotypes and multicellular patterns in brain cancer. *Journal of Theoretical Biology* 244, 96–107. doi:10.1016/j.jtbi.2006.06.034

Zheng, J. (2012). Energy metabolism of cancer: Glycolysis versus oxidative phosphorylation (review). *Oncology Letters* 4, 1151–1157. doi:10.3892/ol.2012.928

Zu, X. L. and Guppy, M. (2004). Cancer metabolism: facts, fantasy, and fiction. *Biochemical and Biophysical Research Communications* 313, 459–465. doi:10.1016/j.bbrc.2003.11.136
